# Light-induced mechanical response in crosslinked liquid-crystalline polymers with photoswitchable glass transition temperatures

**DOI:** 10.1038/s41467-018-05744-x

**Published:** 2018-08-13

**Authors:** Youfeng Yue, Yasuo Norikane, Reiko Azumi, Emiko Koyama

**Affiliations:** Electronics and Photonics Research Institute, National Institute of Advanced Industrial Science and Technology (AIST), Higashi 1-1-1, Tsukuba, Ibaraki 305-8565 Japan

## Abstract

Energy conversion of light into mechanical work is of fundamental interest in applications. In particular, diligent molecular design on nanoscale, in order to achieve efficient photomechanical effects on macroscopic scale, has become one of the most interesting study topics. Here, by incorporating a “photomelting” azobenzene monomer crosslinked into liquid crystalline (LC) networks, we generate photoresponsive polymer films that exhibit reversible photoswitchable glass transition temperatures (*T*_g_) at room temperature (~20 °C) and photomechanical actuations under the stimulus of UV/visible light. The *trans*-to-*cis* isomerization of azo chromophores results in a change in *T*_g_ of the crosslinked LC polymers. The *T*_g_ of the polymer network is higher than room temperature in the *trans*-form and lower than room temperature in the *cis*-form. We demonstrate the photoswitchable *T*_g_ contribute to the photomechanical bending and a new mechanism for photomechanical bending that attributes the process to an inhomogeneous change in *T*_g_ of the film is proposed.

## Abstract (web summary)

Energy conversion of light into mechanical work is of fundamental interest for a wide range of applications, but the development of efficient light responsive polymers is challenging. Here the authors demonstrate photoswitchable  glass transition temperatures contribute to the photomechanical bending in a crosslinked azobenzene polymer.

## Introduction

The development of new materials that can change reversibly in response to external stimuli, such as light, has attracted significant attention from researchers owing to their potential applications in many fields. Light is a clean energy source and a non-contact stimulus, which can be used specifically with regard to wavelength (in the ultraviolet (UV) to infrared (IR) region), intensity, or polarization direction to achieve precise and remote control^1–3^. Light-responsive materials have various applications in photonics, e.g., in optical switching^4–6^, data storage^7,8^, lasing^9,10^, and actuations^11–18^.

Light-responsive materials in which a quick and reversible response can be achieved are of particular interest. However, the development of highly photoresponsive polymers that can convert light into mechanical work in an efficient manner remains a research challenge, which hampers their range of applicability. The difficulty in realizing efficient photomechanical actuations in solid matrices can be attributed to the fact that the performance of functional photochromic molecular switches, such as azobenzene or spirooxazine, is highly sensitive to the viscosity of their polymer matrix. Using fluid environments or lowering the glass transition temperature (*T*_g_) of the polymer matrix can improve the switching speeds^19–21^ but can compromise other physical properties such as hardness. Thus, a material that can temporarily or reversibly change its *T*_g_ under light stimuli is preferable.

For photoresponsive materials, a few azobenzene compounds, that undergo a solid-to-liquid phase transition under photoinduced *trans*-to-*cis* isomerization have been reported^22–24^. Most of these compounds required prolonged exposure to strong UV irradiation (e.g., exposure for 30 min to a light intensity of 40–100 mW cm^−2^) to trigger such a transition^23–26^. Designing new molecules that have high photosensitivity (i.e., the solid-to-liquid phase transition occurs within several seconds at a moderate light intensity) remains difficult. Furthermore, the design of such molecular switches with polymerizable groups, such as monomers, is essential for the development of photoactive polymers, rubbers, or gel materials. In contrast to small molecules showing solid-to-liquid phase transitions, polymers exhibit *T*_g_ values that determine whether they are glass-like or rubber-like. Polymers with a lower *T*_g_ are more flexible and softer at the temperature of their application, and thus respond quickly to mechanical processes such as bending.

To date, little attention has been paid to polymers with photoswitchable *T*_g_. Recently, the Wu group found a non-crosslinked azo polymer that shows photoswitchable *T*_g_ and reversible solid-to-liquid phase transitions^27^. However, photoswitchable *T*_g_ in a crosslinked polymer has not been reported. Moreover, the deeper relationship between photomechanical actuations and photoswitchable *T*_g_ in azo polymers has never been demonstrated.

**Fig. 1:**
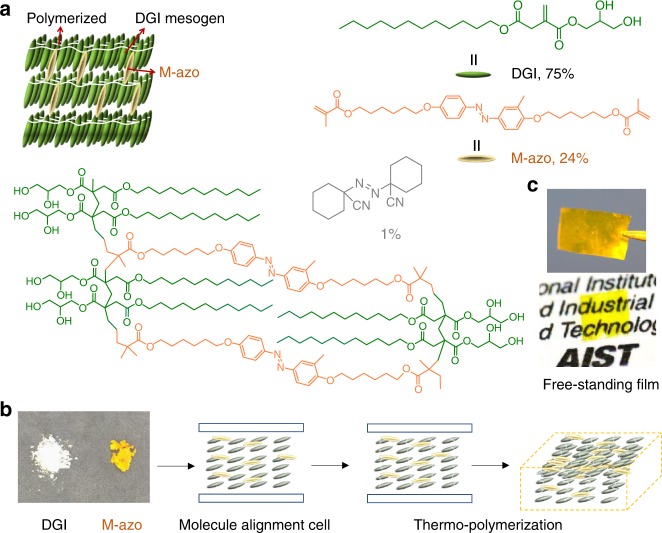
Schematic representations of molecular structures and their packing modes in the polymer film. **a** The crosslinked polymer network structure in the film. **b** Schematic illustration of the fabrication procedure of the polymer film in a molecule alignment cell to realize macroscopic ordering in the film. **c** An as-prepared free-standing film that shows high transparency

In this work, we develop a photoresponsive film based on a LC network using a polymerizable *meta*-methylazobenzene (M-azo) as the photoresponsive moiety that was covalently crosslinked into a dodecyl glyceryl itaconate (DGI) polymer network (Fig. 1). The free-standing LC film shows a change in its glass transition temperature and reversible photomechanical motions upon exposure to UV/visible light. We found that the *T*_g_ of the LC polymer network decreases after UV absorption; as soon as the UV irradiation is removed, the polymer network recovers its *T*_g_ at room temperature upon exposure to visible light or in the dark. The *trans*-to-*cis* photoisomerization of azo that crosslinked in the polymer network results in a photoswitchable *T*_g_. We demonstrate the reversible change in the *T*_g_ of the polymer network contribute to the photomechanical response (e.g. bending) in LC film. Moreover, a mechanism for photomechanical response due to the inhomogeneous *T*_g_ change in azo polymer film is proposed.

## Results

### Fast photomelting of M-azo monomer at room temperature

**Fig. 2:**
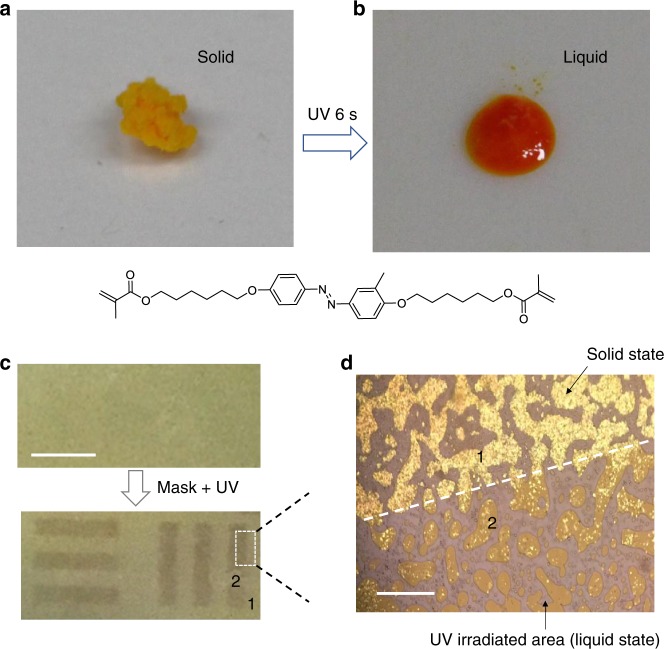
Photographic images of the M-azo compounds that show photomelting under UV light. **a**, **b** M-azo compounds before (**a**) and after (**b**) UV irradiation (*λ* = 365 nm, 125 mW cm^−2^) at room temperature. **c** A solid-to-liquid phase transition can be observed in the UV-irradiated areas, whereas the regions not irradiated (covered from UV exposure by using a metal mask) retained the solid state. Scale bar, 5 mm. **d** Electron microscope image clearly revealing the boundary (dashed-dotted line) between the solid and liquid states in M-azo. Scale bar, 100 μm

The solid-to-liquid phase transition is one of the most fundamental phenomena in material science. It is a process in which the intermolecular forces, such as van der Waals, present in the solid state weaken at a certain temperature or pressure. However, little attention has been given to the “photomelting” phenomenon despite a rich functionality of these photomelting compounds^23,28^. It is therefore exciting to discover that M-azo compounds exhibit rapid photomelting under light irradiation at room temperature. As shown in Fig. 2a, b, the compounds change from a solid to liquid state within 6 s upon irradiation with UV light (*λ* = 365 nm) at an intensity of 125 mW cm^−2^. By using a metal mask on M-azo under illumination, it was observed that the irradiated regions converted to the liquid phase, while the mask-covered regions retained the solid phase. A clear pattern/boundary demarcating the two regions can be observed both on the macroscale (Fig. 2c) and microscale (Fig. 2d).

The photomelting process is closely related to molecular packing and weakening of the intermolecular forces during photon absorption^29,30^. As detected under polarized optical microscopy (POM), the M-azo crystals showed bright birefringence patterns before UV irradiation, which indicated an ordered packing of the molecules at room temperature (Supplementary Fig. 1). Upon UV irradiation, the birefringence of the compounds disappeared quickly, which corresponded to the change from crystal phase to isotropic phase (Supplementary Fig. 2). In this process, the basic stereochemical conversion of M-azo molecules is from the extended *trans* to *cis*-isomers and involves a change in molecular length from 9 to 5.5 Å^31^. Generally, the photoisomerization process is not accompanied by a solid-to-liquid phase transition. However, for this compound, fast photomelting is observed, which indicates the rapid weakening of intermolecular cohesive forces during photon absorption^30^. This quick phase transition is mainly attributed to *meta*-substitution in the azo molecular unit, which may significantly change the molecular packing and increase the intermolecular distance (to trigger the solid to liquid change) upon UV irradiation.

To test this, we also synthesized a more common non-substituted azo molecule in which the methyl group (of M-azo) was replaced with hydrogen (denoted as H-azo, Supplementary Fig. 3). In contrast with M-azo, H-azo showed no photomelting under identical conditions of light irradiation. As shown in Supplementary Movie 1 and Supplementary Fig. 4, the M-azo and H-azo molecules were placed close to each other with dot sizes of 1−2 mm on a slide glass and observed by POM at room temperature. Once the UV light (*λ* = 365 nm, intensity = 80 mW cm^−2^, illumination area = 2×2 cm) was turned on, the M-azo region (right) displayed fast photoinduced melting with disappearing birefringence, whereas the H-azo molecule (left) revealed no change. The photoinduced solid-to-liquid phase transition was not a photothermal process but was due to the photoisomerization, because UV light with an intensity of 80 mW cm^−2^ for 10 s cannot increase the surface temperature to the melting temperature of M-azo (65 °C) or H-azo (73 °C) (Supplementary Fig. 5).

To further evaluate the photosensitivity of the M-azo and H-azo molecules, we used a POM system equipped with an optical spectrophotometer to monitor the kinetics of photochemical phase transitions^32^. As the sample was irradiated with UV light during the experimental measurement, a profile of the kinetic loss in birefringence was simultaneously obtained by the spectrophotometer (Supplementary Fig. 6). The transmittance fade speed is denoted as *T*_1/2_, and is defined as the time taken for the optical density of the specimen to decrease to one-half of its initial value. In this study, the fade in transmittance speeds was fast for M-azo (*T*_1/2_ ~ 3 s), while H-azo showed no decrease in transmittance during the testing time. The phase transition in M-azo was also confirmed by a 3D laser scanning electron microscope. As shown in Supplementary Fig. 7, the material changes from a solid to liquid state under UV irradiation. To the best of our knowledge, there are no other reports on a polymerizable monomer that can show fast photoinduced solid to liquid phase transition.

### Fabrication of free-standing films

**Fig. 3:**
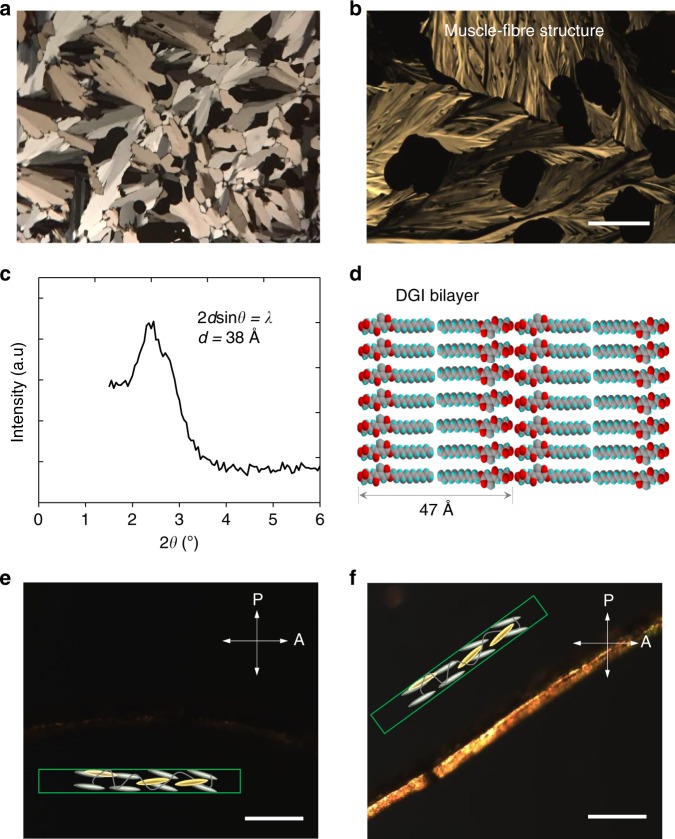
Structural characterization of DGI monomers and the polymer films. **a** DGI monomer revealing anisotropic aggregations with a liquid crystal texture under POM. **b** The patterns in the DGI liquid crystals look similar to those of a muscle-fiber structure under POM. Scale bar, 100 μm. **c** X-ray diffraction pattern of the free-standing polymer film at room temperature that shows a peak at about 2*θ* = 2.3°, corresponding to bilayer distances (*d*) of ~38 Å. **d** The molecular length of the DGI bilayer shown in the fully extended conformation is about 47 Å. The cross-section of the film was tilted at **e** 0° and **f** 45° angles relative to the transmission axis of the analyser under POM. The white bidirectional arrows on the top right represent the directions of the analyser (A) and polarizer (P). Scale bars, 30 μm

We fabricated a free-standing polymer film by free-radical polymerization of M-azo and DGI. DGI was synthesized according to the literature;^33^ it has been previously used in functional hydrogels^34–37^. The DGI molecules displayed smectic LC textures when observed using a POM system (Fig. 3a). Smectic phase is LC phase with a layered structure, which are more ordered than the nematic phase. Three main types of nematic LC monomers have been typically used within the last 10 years to synthesize the photoresponsive polymers that are employed in photomechanical LC films (Supplementary Table 1). This is probably because developing a new polymerizable LC monomer that is applicable to photomechanical polymers is not easy. In this study, the DGI monomer was initially used as a candidate to serve as a muscle-like mesogen moiety that attached as a side chain onto the polymer backbones (Fig. 1a and  Fig. 3b). DGI shows no photoresponsive property (Supplementary Fig. 8); the M-azo displays high photosensitivity and serves as a crosslinker. The DGI monomers can self-assemble into lamellar structures due to the presence of the LC phase, and the long alkyl chain (C-12) in DGI and M-azo monomers may enable sufficient free volume to generate a distinct photomechanical response^38^.

During synthesis, the pre-heated precursor mixture containing DGI (22 mg), M-azo (7 mg), and initiator (0.3 mg) was pulled into a molecule alignment cell (area = 2 × 2 cm, spacing = 5 or 10 μm) by capillary pressure (Fig. 1b; for details on synthesis, see Methods). The cell was placed on a hotplate at 60 °C for 1 h and then at 125 °C for 24 h. After cooling to room temperature, the cell was opened with knives and the film was detached from the cell with tweezers, yielding a transparent free-standing film with a thickness of 5 or 10 μm, depending on the spacer thickness (Fig. 1c). The film (denoted as DGI/M-azo) was characterized using X-ray diffraction (XRD) analysis. The peak in the XRD spectrum at 2*θ* = 2.3° indicates that the layer spacing of the lamellar structure was about 3.8 nm, which was smaller than the calculated molecular length of the bilayer (~4.7 nm) in the fully extended conformation^33,34,39^ (Fig. 3c, d). This indicates that DGI was probably in the smectic C LC phase. To further investigate the molecular orientation along the thickness direction, we also characterized the film using POM. The images revealed a clear contrast for a 45° angular rotation, whereas they were dark when the angle between the incident light and the sample was 0° or 90°, which implies that the mesogenic groups had a unidirectional molecular orientation (Fig. 3e, f).

### Photoswitchable *T*_g_ films

**Fig. 4:**
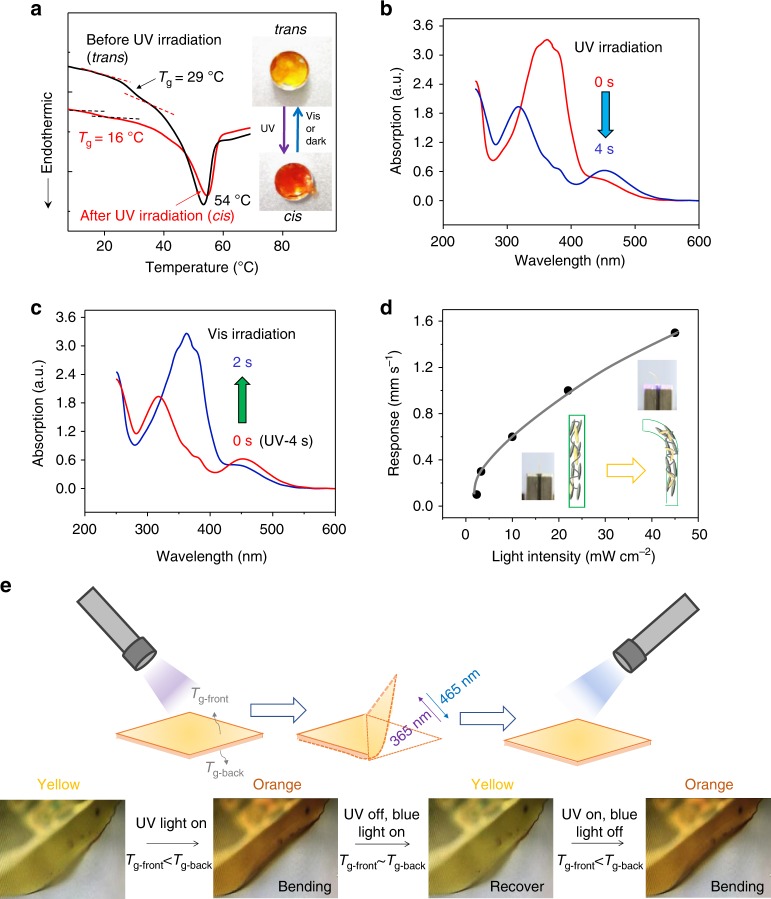
Photoswitchable *T*_g_, UV/Vis absorption spectra and photomechanical response of the film. **a** DSC measurements of *T*_g_ during the first heating process of the polymer in *trans*-, and *cis*-form. **b** Absorption spectra of a DGI/M-azo film before (red line) and after (blue line) irradiating with a light of *λ* = 365 nm for 4 s (light intensity = 100 mW cm^−2^). **c** Absorption spectra of a DGI/M-azo film before (red line) and after (blue line) irradiation using light of *λ* = 465 nm for 2 s (light intensity = 100 mW cm^−2^). **d** The relationship between the photoresponse speed of the fresh DGI/M-azo fiber and UV light intensity. **e** The continuous photomechanical response of the polymer film under light irradiation. The UV irradiation (*λ* = 365 nm, 11 mW cm^−2^) caused bending of the film; the visible light (*λ* = 465 nm, 30 mW cm^−2^) subsequently straightened the bent film. *T*_g-front_, *T*_g_ value of the film on the front side; *T*_g-back_, *T*_g_ value of the film on the backside

The *T*_g_ exists in both amorphous polymers and crystalline polymers. Generally, the *T*_g_ of a polymer is related to the temperature at which its molecular chains start to move, and cannot change once the composition and structure of the polymer is fixed. Because of the photoinduced rapid phase transition of M-azo monomers covalently crosslinked into the polymer network, the *T*_g_ of the polymer can be reversibly switched under UV/visible light irradiation. Such polymers with photoswitchable *T*_g_ were not experimentally demonstrated in literature until very recently^27^. Wu et al. found that a non-crosslinked azo polymer shows a change in *T*_g_ from 54 to −10 °C after UV irradiation. In this work, DGI/M-azo polymer is a crosslinked polymer network (Supplementary Fig. 9). We measured the *T*_g_s of the films using differential scanning calorimetry (DSC). As shown in Fig. 4a, the *T*_g_ of the DGI/M-azo polymer decreased from 29 (before UV irradiation) to 16 °C after UV irradiation. The *trans*-form of the DGI/M-azo films exhibited a higher *T*_g_ (29 °C) than room temperature, while the same film in the *cis*-form shows a lower *T*_g_ (16 °C) after UV irradiation. For details on the DSC measurement, the DGI/M-azo films (~5 μm, 8 mg) were cut into small pieces and transferred to the DSC crucible (Supplementary Fig. 10). After measurement, the films were taken out of the crucible and hold for overnight. On the second day, the same pieces of films were irradiated with UV light (intensity = 125 mW cm^−2^) on both sides for 2 min and transferred to a new crucible. The *T*_g_ was measured in the same films with *cis*-form. From the DSC curves, above *T*_g_ there is another endothermic peak, which could be attributed to the phase transition of the fundamental components, DGI mesogens (Supplementary Fig. 11). This peak was not detected on the second heating, which was done immediately after first heating and cooling (Supplementary Fig. 12). But this peak appears again after holding the sample overnight, suggesting that the rearrangement of the polymer chains is slow^40^. The change in *T*_g_ under UV irradiation was accompanied by an obvious color change of the film from pale yellow to orange, as shown in Fig. 4a (inset).

The degree of change in *T*_g_ is determined by the *trans* to *cis* ratio in the azo units in the polymer. For a pristine non-crosslinked azo polymer, *T*_g_ was decreased by ~60 °C when more than 90% of the polymer was in the *cis*-azo form^27^. In our case, the UV irradiation cannot completely isomerize all the *trans* M-azo into *cis*-isomers, especially in the film state. There are two main reasons for this. First, the conversion under continuous UV exposure reaches the photostationary state of azobenzene. The photostationary state of 365 nm exposure of M-azo (in CDCl_3_) is calculated to be 97% by nuclear magnetic resonance (Supplementary Fig. 13). Second, in the film state, the strong absorption of *trans*-azo at 365 nm allowed the top layer of the film in particular to be converted to *cis*-azo. The conversion of covalently embedded azobenzene in DGI/M-azo film is ~86% of *trans* isomerized into *cis* after UV irradiation for 35 s at an intensity of 40 mW cm^−2^ (Supplementary Fig. 14). Thus, the weight content of *cis*-isomers in the whole film (photostationary state of 365 nm exposure) is close to 21 wt% as the total azo content in the film is 24 wt%.

More samples were measured to characterize the *T*_g_ change in DGI/M-azo films before and after UV irradiation (Supplementary Fig. 15). Moreover, the *T*_g_ value is closely related to the mechanical properties of the polymer films. A higher *T*_g_, along with a high storage modulus, results in a high stiffness, which equates to a low percentage elongation when stressed. We then measured the mechanical properties of the DGI/M-azo film before (*trans*-form) and after UV irradiation (*cis*-form) by tensile tests (Supplementary Fig. 16). The polymer film before UV irradiation showed a Young’s modulus as high as 140 MPa. However, the polymer film after UV irradiation (*cis*-form), showed a much lower Young’s modulus, 65.6 MPa but a higher percentage elongation (strain) (Supplementary Fig. 16). This also indicates that the UV light decreased the *T*_g_, and thus decreased the Young’s modulus of the polymer films.

### Photomechanical response in photoswitchable *T*_g_ films

Most of the reported photomechanical response times for bulk solid materials (e.g., LC polymers) exposed to strong UV irradiation are on the order of seconds to minutes. For example, the films fabricated using a common H-azo and LC monomers displayed a slow response, ~30 s for a UV light intensity of 500–850 mW cm^−2^ with a UV irradiation dose (time × intensity) of 15–25 s W cm^−2^^41^, or several minutes for an intensity of 60–170 mW cm^−2^ (UV irradiation dose = 3.6–10.4 s W cm^−2^)^13,42^. Hosono et al. reported on a fast, non-crosslinking polymer actuator that required UV irradiation for 10 s at an intensity of 300 W to trigger a photomechanical displacement of 2 mm with a UV irradiation dose of 3000 s W cm^−2^^43^. One of the factors that strongly affects the response time of polymer films is a high *T*_g_^21^. Decreasing the *T*_g_ or increasing the softness of the polymer may improve its response time.

As observed above, we found that the *T*_g_ value of the DGI/M-azo polymer can be decreased upon UV irradiation and recovered in the dark or by visible light irradiation. The photoresponse of the film was quick which was confirmed by UV/vis spectroscopy. As shown in Fig. 4b, c, the fast decreasing in the absorbance at 365 nm under UV light and even faster back isomerization process under visible light finished within 2–4 s. The back thermal isomerization is slow in the dark (Supplementary Fig. 17).

The corresponding photoswitchable *T*_g_ is beneficial for achieving efficient photomechanical actuations in the polymer network. To further characterize this phenomenon, we inspected the photomechanical response of the DGI/M-azo polymer. As shown in Supplementary Movie 2, the bending and recovery of the polymer film can be repeated by switching on/off the UV and visible light continuously. When the UV light (*λ* = 365 nm) was switched on, the film bent in the direction of the irradiation at the same time, whereas when the blue light (*λ* = 465 nm) was turned on, the film was restored to its flat (original) state immediately. The bending of the film was accompanied by a color change from yellow to orange and then back (orange to yellow). Here, the bending/unbending processes of the film could be controlled and repeated for many cycles without any obvious fatigue (Supplementary Movie 2).

Photoabsorption is a time-dependent process, and the degree of change in *T*_g_ and the response speed should be dependent on the light intensity. To characterize the relationship between the light intensity and the response speed, we applied different intensities of UV light for irradiating a DGI/M-azo polymer fiber. As shown in Fig. 4d and Supplementary Movie 3, the photoresponse speed improved gradually with increasing intensity of the irradiated light. When the light intensity was increased from 3.28 to 45 mW cm^−2^, the bending speed (displacement/time) improved from 0.4 to 2 mm s^−1^ at a testing temperature of ~20 °C. For DGI/M-azo film, the average irradiation dose is ~0.046 s W cm^−2^ for a bending displacement of 3 mm. When the film bent to the direction parallel to the light, it stopped bending and started to oscillate^44,45^. Compared with previously reported photoresponsive films that need a relatively large UV irradiation dose, this polymer can efficiently absorb a small number of photons to cause mechanical reactions.

### Mechanism for photomechanical response

XRD analysis and Fourier transform infrared (FT-IR) spectroscopy were employed to study the structural changes occurring inside the polymer during photoabsorption and characterize the film before and after UV irradiation. As shown in Supplementary Fig. 18, there was no obvious change in the peak position and intensity in the XRD pattern or IR spectrum, indicating that the order parameter of the structure had not been significantly altered. In such situations, bending cannot be explained by using the generally reported mechanism alone, which attributes the process to a reduction in the order parameter of the films^46^. Considering these observations, we propose a complementary mechanism for photomechanical bending that attributes the process to an inhomogeneous change in *T*_g_ of the film (Fig. 4e). Absorption of photons first occurs on the front surface of the film, and then, the residual photons are absorbed in the inner region, which results in a difference in *T*_g_ values between the front and back surfaces of the film, i.e., *T*_g-front_ < *T*_g-back_. Thus, a difference in the internal strains, i.e., *Ɛ*_front_ < *Ɛ*_back_, is produced, causing the film to bend in the direction of the incident UV light.

## Discussion

In this work, M-azo compounds showed photomelting within several seconds, exhibiting their ability to quickly overcome the intermolecular forces^30^ during *trans*-to-*cis* isomerization. In the *trans* state, the intermolecular forces are high, such that M-azo exists as a solid; in the *cis* state, it is liquid due to the intermolecular forces being overcome after UV irradiation. The value of *T*_g_ is greatly influenced by the intermolecular forces^47^. Jenekhe et al. reported a direct experimental observation of the effects of intermolecular forces on the *T*_g_ of diverse polymers and showed that alleviation of intermolecular forces causes a dramatic shift of the *T*_g_ to a lower temperature^47^. Similar to small molecules, the *trans* and *cis* configuration azopolymers may have different intermolecular forces, which influence the *T*_g_ values.

When the temperature during use, *T*_using_, is lower than the *T*_g_ of the polymer (i.e., *T*_g_ > *T*_using_), the polymer is in a relatively glassy state. When *T*_g_ of the polymer is decreased via specific photon absorption to a value lower than the use temperature (i.e., *T*_g_ < *T*_using_), the polymer is in a rubber state and response speed of the polymer is probably improved.

Moreover, we think the design of highly photoresponsive polymers should first begin from a basic monomer design, because certain properties observed at the molecular level (e.g., fast photomelting during photoisomerization) may be amplified in the polymeric materials with interesting macroscopic properties, such as a high rate of change of *T*_g_ by photoswitching. Second, by coupling the highly photoresponsive molecular switches covalently embedded in the ordered parameter of the LC networks (i.e., DGI), the collective molecular motion in ordered materials is amplified^11^.

In summary, we reported the light-induced mechanical response of a LC polymer that show photoswitchable *T*_g_. We used an azo crosslinker, M-azo, which shows photoinduced melting, to crosslink the LC monomer DGI. The azo chromophores in the polymer network showed reversible *trans*-to-*cis* photoisomerization, resulting in the *T*_g_ change of the crosslinked polymer network. The polymer network had a *T*_g_ higher than room temperature in the *trans*-form and lower than room temperature in the *cis-*form. We further studied the photomechanical actuation of the polymer network and demonstrated that photoswitchable *T*_g_ contributes to light-induced bending. Especially, we propose a mechanism for photomechanical response due to an inhomogeneous change in the *T*_g_ of the film. We anticipate these results provide a deeper insight in the photomechanical response in azobenzene LC polymers.

## Methods

### Materials and analytical methods

The solvents, tetrahydrofuran (THF), *N*, *N*-dimethylformamide (DMF), toluene, dimethyl sulfoxide-d6 (DMSO-d6), and deuterated chloroform, (CDCl_3_) were purchased from Aldrich and used as-received. The thermal initiator 1,1′-azobis(cyclohexane-1-carbonitrile) (V-40, chemical structure shown in Fig. 1) was purchased from Wako Pure Chemical Industries Ltd., Japan. Silica gel (40 μm) was also purchased from Wako Pure Chemical Industries Ltd., Japan, and used in column chromatography. More details about the materials are shown in the Supplementary Note 1. ^1^H NMR and ^13^C NMR spectra were measured using Bruker Advance NMR spectrometers at resonance frequencies of 400 MHz and 500 MHz, respectively. Multiplicities are abbreviated as follows: singlet (s), doublet (d), triplet (t), and multiple (m). High-resolution mass spectrometry (HRMS) was measured using a JEOL spiral TOF JMS-S3000 spectrometer. The light source used was an LED lamp (CCS, HLV-24VV365-4W PCLTL for *λ* = 365 nm; HLV2-22βL-3W for *λ* = 465 nm), or a high-power mercury light (REX-250) with different filters. The light intensity was monitored by a Newport 1917-R optical power meter with 818-ST-UV photodetector or USHIO-accumulated UV meter UIT-250. The surface temperature of the compounds (M-azo) before and after UV irradiation was measured by an infrared thermometer (TG167, FLIR). The 3D laser scanning electron microscopic images were obtained using a Keyence VK-X100 laser microscope with a laser wavelength of 658 nm. DSC thermograms were obtained using SII Nanotechnology DSC6100. More details about the methods are shown in the Supplementary Methods.

### Monomer synthesis and characterization

The detailed organic synthesis procedures of M-azo, H-azo, intermediate products, DGI monomer, and their structural characterizations are shown in the Supplementary Note 1.

### Film fabrication

LC polymer films were fabricated by copolymerizing M-azo and DGI monomers via thermal initiation. A mixture of the monomers containing 22 mg DGI, 7 mg M-azo, and 0.3 mg initiator was melted by heating at a temperature of 70 °C in a vial. A small amount of toluene (20 μl) was added to decrease the viscosity of the mixture. The mixture was drawn by capillary pressure into a parallel setup containing molecule alignment cells (thickness = 5 μm or 10 μm, area = 2×2 cm, KSRP-50/A107P1NSS or DONNELLY, E.H.C Co. Ltd), which were pre-heated to 70 °C. For polymerization, the sample was heated at 60 °C for 1 h on a hot plate, followed by heating to 125 °C for 24 h under a N_2_ atmosphere to ensure a full conversion of the acrylate monomers.

The entire process was carried out in a lab with <500 nm cut-off light. After polymerization, the cell was opened with knives and the DGI/M-azo films were removed from the glass substrates with tweezers.

### Film characterization

The molecular alignment of the films was studied by observing their cross-section using an Olympus BX51 POM equipped with a Linkam heating stage. Absorption spectra were recorded on a JASCO V-670 using double-beam spectrophotometer. The XRD spectra were obtained using a SmartLab Rigaku X-ray analytical machine with a Cu Kα (*λ* = 1.5418 Å) source. Tensile stress–strain curves were acquired using a commercial machine (Tensilon EZ-LX, SHIMAZU). FT-IR spectra were recorded on a Perkin-Elmer Spectrum 2000 spectrometer in the wavelength region of 370–4000 cm^−1^. More detailed descriptions of the characterization methods can be found in the Supplementary Methods.

### Data availability

The authors declare that the main data supporting the findings of this study are available within the article and its Supplementary Information files, or available from the corresponding author upon reasonable request.

## Electronic supplementary material

Supplementary Information

Description of Additional Supplementary Files

Supplementary Movie 1

Supplementary Movie 2

Supplementary Movie 3
