# Supplementary material for: Light-induced mechanical response in crosslinked liquid-crystalline polymers with photoswitchable glass transition temperatures
Source: Nat Commun. 2018 Aug 13;9:3234. doi: 10.1038/s41467-018-05744-x (PMC6089925; doi:10.1038/s41467-018-05744-x)
Supplement: Supplementary file 1 — Supplementary Information [file 41467_2018_5744_MOESM1_ESM.pdf]

Supplementary Information

Light-induced mechanical response in crosslinked liquid-crystalline polymers with photoswitchable glass transition temperatures

Yue et al

Table of Contents

Supplementary Note 1

Materials; synthesis of azo monomers (M-azo, H-azo); synthesis of DGI liquid-crystalline monomers

Supplementary Methods

^{1}H NMR or ^{13}C NMR spectra; Polarized optical microscopy; 3-D scanning electronic microscopy; Differential scanning calorimetry; UV–Vis absorption spectra; X-ray diffraction; Fourier transform infrared spectroscopy

Supplementary Figures

Supplementary Figure 1. The detection of M-azo molecules' phase transition under polarized optical microscopy (POM).

Supplementary Figure 2. The POM detection of the quick photomelting process of M-azo under UV irradiation.

Supplementary Figure 3. The chemical structures of H-azo and M-azo

Supplementary Figure 4. Difference in the photomelting properties of M-azo and H-azo placed together on a slide glass under polarized optical microscopy.

Supplementary Figure 5. Surface temperature of the compounds (M-azo) before and after UV irradiation measured using an infrared thermometer.

Supplementary Figure 6. Evaluation of kinetics of photoinduced phase transition for M-azo and H-azo.

Supplementary Figure 7. The 3D scanning electronic microscopy shows the UV irradiation induced a solid-to-liquid state change of M-azo.

Supplementary Table 1. Nematic liquid-crystalline monomers that typically used in photoresponsive polymers in literature.

Supplementary Figure 8. The POM images of DGI compounds at UV (λ = 365 nm) irradiation for different time.

Supplementary Figure 9. An experiment shows that the M-azo is covalently crosslinked in the polymer network.

Supplementary Figure 10. The detail process on T_g measurement of the *cis*-, and *trans*-film.

Supplementary Figure 11. Differential scanning calorimetry (DSC) curves and POM images of M-azo under heating and cooling.

Supplementary Figure 12. The DSC curves of the DGI/M-azo film before and after UV irradiation at the second heating.

Supplementary Figure 13. ^{1}H NMR spectra of M-azo in $CDCl_3$ before and after 365 nm irradiation.

Supplementary Figure 14. *Cis*-contents of M-azo in the solution and film state.

Supplementary Figure 15. DSC first heating curves of the DGI/M-azo films in *cis*-, *trans*-form.

Supplementary Figure 16. Stress-strain curves of DGI/M-azo films before (*trans*-form) and after UV irradiation (*cis*-form).

Supplementary Figure 17. UV-vis absorption spectra of thermal relaxation of the *cis* state to *trans* state of DGI/M-azo film measured at room temperature in the dark.

Supplementary Figure 18. Structural characterizations of the same film before and after UV irradiation.

Supplementary References

Supplementary Note 1

Materials

Chemicals: Itaconic anhydride was purchased from Sigma Aldrich without further purification. Dodecanol was received from TCI (Japan), 1,1'-Azobis(cyclohexane-1-carbonitrile) (V-40) was purchased from Wako Pure Chemical Industries Ltd., Japan. The slide glass (Matsunami Glass Ind., Ltd. Square microscope cover glass No.1, 18 mm × 18 mm, thickness: 0.12-0.17 mm), were used as the glass substrate for the optical observations.

Synthesis of azo monomers

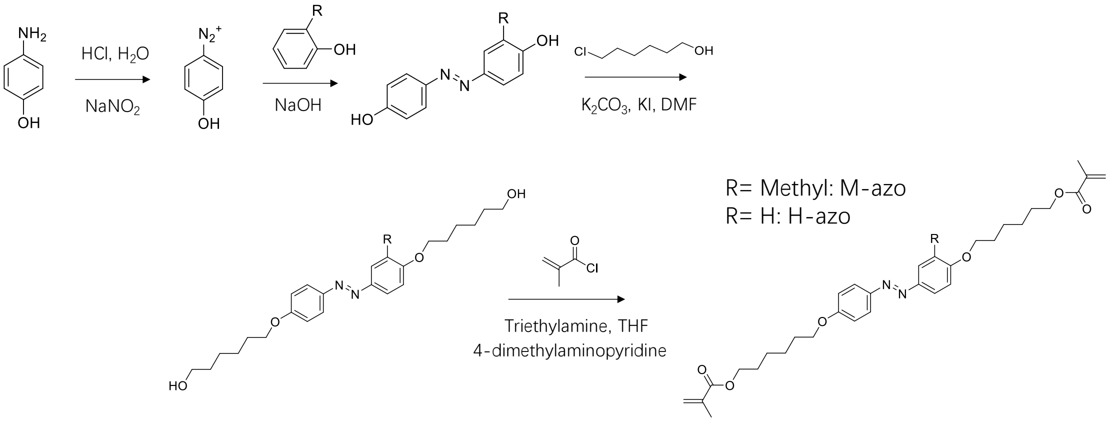

Synthesis of 4,4'-Dihydroxy-3-methlazobenzene (**1a**)

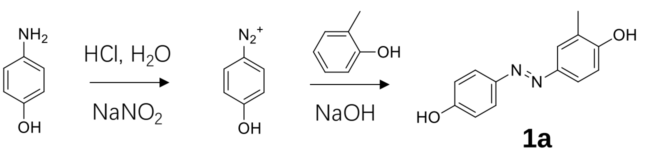

1a

4,4'-Dihydroxy-3-methlazobenzene (**1a**) was synthesized via a typical azo-coupling reaction. 4-aminiphenol (4.36 g, 40 mmol) was dissolved in a diluted hydrochloric acid solution (2.4 N, 50 mL) and the solution was kept at -3 °C. Sodium nitrite (3.32 g, 48 mmol) in water (4 mL) was dropwise into the solution to produce diazonium salt. The mixture was stirred for 30 min and added dropwise to a solution of o-cresol (4.32 g, 40 mmol) in sodium hydroxide solution (20%, 16 mL) at -3 °C and then a yellow solid precipitated. The mixture was stirred at room temperature for 22 h. After hydrochloric acid (2.4 N) was added to neutralize the reaction mixture. The brown solid was filtered, washed with water, and dried. The crude material was purified by column chromatography on silica gel (eluent: ethyl acetate/n-hexane (1:2)) and recrystallization from acetone/n-hexane to give 4,4'-a yellow crystalline solid (Compound **1a**). ^{1}H NMR (400 MHz, DMSO-d6) δ 10.08 (s, 1H), 10.04 (s, 1H), 7.70 (d-d, J1 = 6.8 Hz, J2 = 1.9 Hz, 2H), 7.60 (d, J= 2.0 Hz, 1H), 7.55 (d-d, J1 = 8.4 Hz, J2 = 2.4 Hz, 1H), 6.92 (d, J = 8.4 Hz, 1H), 6.90 (d-d, J1 =6.8 Hz, J2 = 1.9 Hz, 2H), 2.20 (s, 3H) ; ^{13}C NMR (125 MHz, DMSO-d6) δ 160.1, 158.4, 145.5, 145.3, 125.1, 124.3, 122.6, 116.0, 115.1, 16.2.

Synthesis of 4,4'-(diazene,1,2-diyl) diphenol (**1b**)

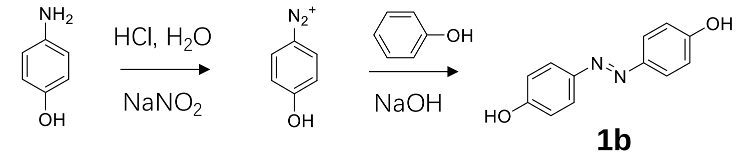

Compound **1b**: ^{1}H NMR (400 MHz, DMSO-d6) δ 10.12 (s, 2H) 7.72 (d-d, J1=3.08 Hz, J2=2.08 Hz, 2H), 7.70 (d-d, J1=2.16 Hz, J2=3.04 Hz, 2H), 6.92 (d-d, J1=3.12 Hz, J2=2.08 Hz, 2H), 6.89 (d-d, J1=2.12 Hz, J2=3.12 Hz, 2H). ^{13}C NMR (100 MHz, DMSO-d6) δ 159.98, 145.25, 124.14, 115.78

Synthesis of 6-(4-((4-((6-hydroxyhexyl) oxy)-3-methylphenyl) diazenyl) phenoxy) hexan-1-ol (**2a**)

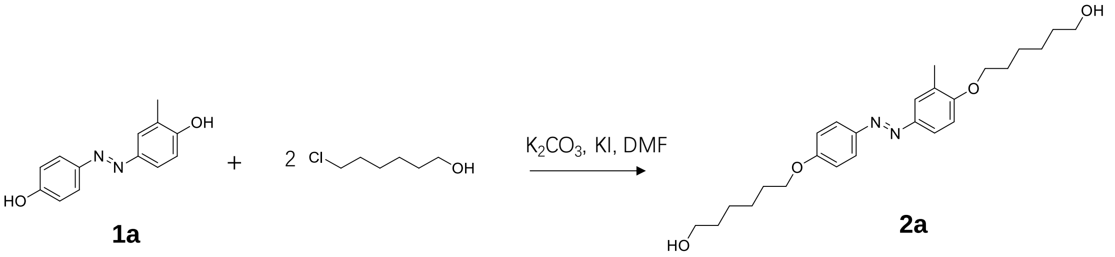

4,4'-Dihydroxy-3-methlazobenzene (2.28 g, 10 mmol), 4.14 g K_2CO_3, and 0.01 g KI was dissolved in 40 mL DMF, and a solution of $HO(CH_2)_6Cl$ (3.0 g, 22 mmol) in 10 mL DMF was dropwise added. The mixture was stirred at 120 °C for 70 h. After the reaction, a large amount of water was added to the mixture to precipitate the product. The crude product was filled off and recrystallized from ethanol to give a yellow powder (compound **2a**). ^{1}H NMR (400 MHz, $CDCl_3$) δ 7.88 (d-d, J1 =6.9 Hz, J2 = 2.0 Hz, 2H), 7.76 (d, J=2.4 Hz, 1H), 7.75 (d, J= 2.7 Hz, 1H), 7.01 (d-d, J1 =6.9 Hz, J2 = 1.9 Hz, 2H), 6.92 (d, J=9.4 Hz, 1H), 4.08 (d-d, J1=11.32 Hz, J2=6.32 Hz, 4H), 3.71 (t, J1=12.52 Hz, J2= 6.16 Hz, 4H), 2.31 (s, 3H), 1.82-1.92 (m, 4H), 1.46-1.68 (m, 12H); ^{13}C NMR (100 MHz, $CDCl_3$) δ 161.39, 159.79, 147.44, 146.83, 127.90, 124.62, 123.85, 115.05, 110.93, 68.54, 68.48, 63.33, 33.11, 29.65, 29.61, 26.39, 26.29, 25.96, 16.80.

Synthesis of 6,6'-((diazene-1,2-diylbis(4,1-phenylene)) bis(oxy))bis(hexan-1-ol) (**2b**)

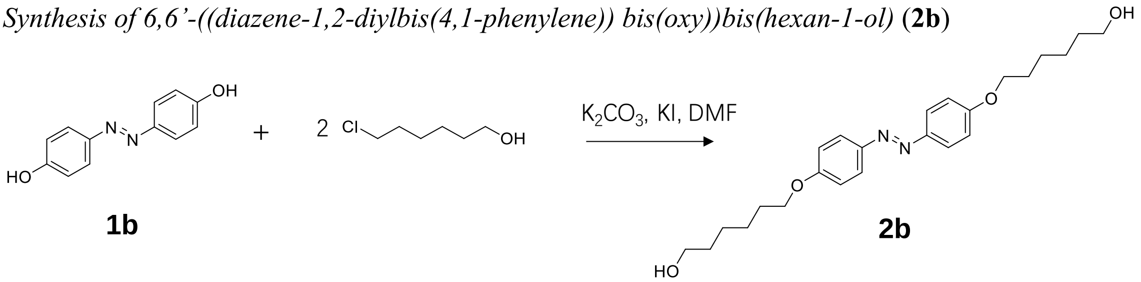

Compound **2b**: ^{1}H NMR (400 MHz, $CDCl_3$) δ 7.87 (d-d, J1=3.12 Hz, J2=2.08 Hz, 2H), 7.84 (d-d, J1=2.08 Hz, J2=3.08 Hz, 2H), 6.99 (d-d, J1=3.12 Hz, J2=1.96 Hz, 2H), 6.97 (d-d, J1=2.12 Hz, J2=3.04 Hz, 2H), 4.04 (d-d, J1=6.48 Hz, 6.48 Hz, 4H), 3.68 (t, J1=6.36 Hz, J2=6.48 Hz, 4H), 1.80-1.87 (m, 4H), 1.40-1.66 (m, 12H)

^{13}C NMR (100 MHz, $CDCl_3$) δ 161.11, 146.96, 124.32, 114.66, 68.16, 62.92, 32. 69, 29.20, 25. 89, 25.56.

Synthesis of 6-(4-((4-((6-(methacryloyloxy) hexyl) oxy)-3-methylphenyl)diazenyl) phenoxy)hexyl methacrylate (**M-azo**)

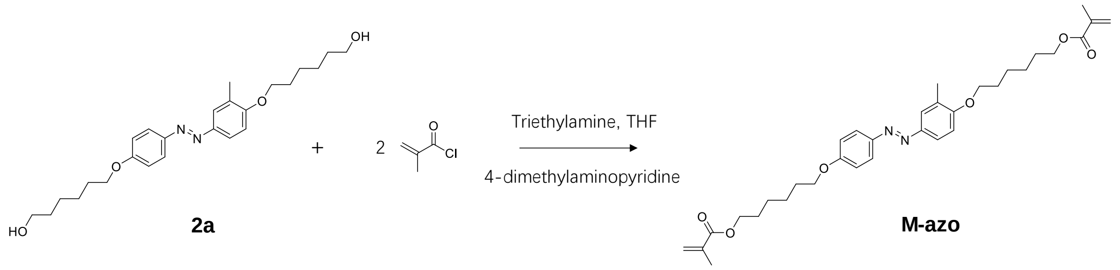

Compound 2a (4.30 g, 10 mmol), triethylamine (1.01 g, 10 mmol), 4-Dimethylaminopyridine (0.280 g) were dissolved in dehydrated THF (260 mL) at 0 °C with stirring. 35 mmol of methacryloyl chloride in dehydrated THF (30 mL) was added to the solution at 0 °C. The reaction was tracked by TLC until most of compound 2a was reacted. The reaction was quenched by pouring water into the mixture. The product was extracted from the solution with chloroform. The chloroform layer was dried with $MgSO_4$. After the solvent was removed, the obtained yellow solid (**M-azo**) was purified by silica gel column chromatography (hexane/ethyl acetate, v/v=10:1) twice.

^{1}H NMR (400 MHz, $CDCl_3$) δ 7.86-7.89 (m, 2H), 7.74-7.77 (m, 2H), 7.02 (d-d, J1 =8.9 Hz, J2 = 3.4 Hz, 2H), 6.93 (d, J=9.4, 1H), 6.12 (s, 2H), 5.57 (s, 2H), 4.18-4.21 (m, 4H), 4.04-4.09 (m, 4H), 2.31 (s, 3H), 1.96 (s, 6H), 1.50-1.88 (m, 16H); ^{13}C NMR (100 MHz, $CDCl_3$) δ 167.95, 159.75, 136.90, 136.85, 125.65, 124.62, 123.87, 123.82, 118.74, 115.04, 110.95, 110.91, 68.48, 68.42, 65.04, 29.56, 29.51, 28.99, 28.96, 26.23, 26.20, 26.15, 18.74, 16.79. HRMS (ESI): m/z $[M+H]^+$ calcd for $C_{33}H_{44}N_2O_6$ (M-azo), 565.3199; found: 565.3194. Melting point = 65 °C.

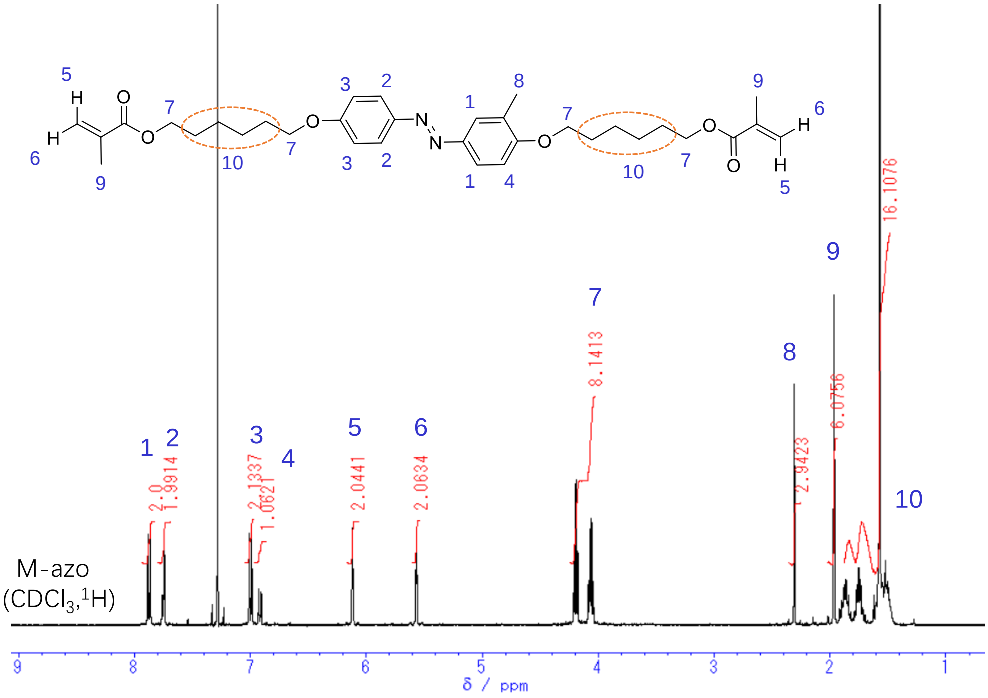

Synthesis of ((diazene-1,2-diylbis(4,1-phenylene)) bis(oxy))bis(hexane-6,1-diyl) bis(2-methylacrylate) (**H-azo**)

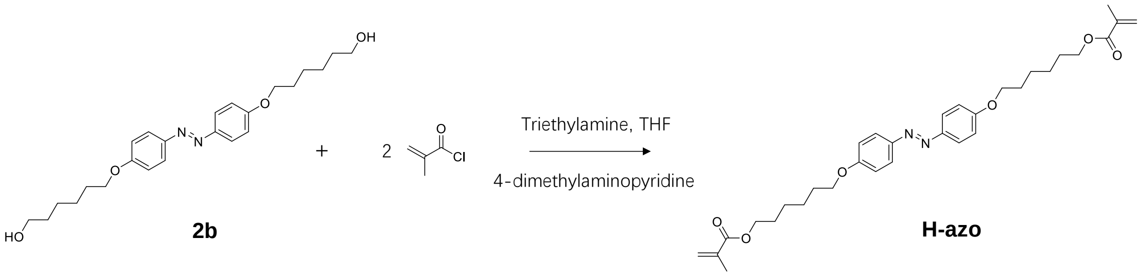

H-azo was reported in the literature previously [1], and it was synthesized via the procedures similar to M-azo. ^{1}H NMR (400 MHz, $CDCl_3$) δ 7.84-7.87 (m, 4H), 6.97-6.99 (m, 4H), 6.12 (m, 2H), 5.55 (m, 2H), 4.15-4.18 (m, 4H), 4.02-4.06 (m, 4H), 1.48-1.96 (m, 22H).^{13}C NMR (100 MHz, $CDCl_3$) δ 161.09, 146.98, 136.51, 125.28, 124.32, 114.65, 102.13, 68.10, 64.66, 29.12, 28.58, 25.83, 25.76, 18.36. Melting point = 73 °C.

Synthesis of DGI liquid-crystalline monomers

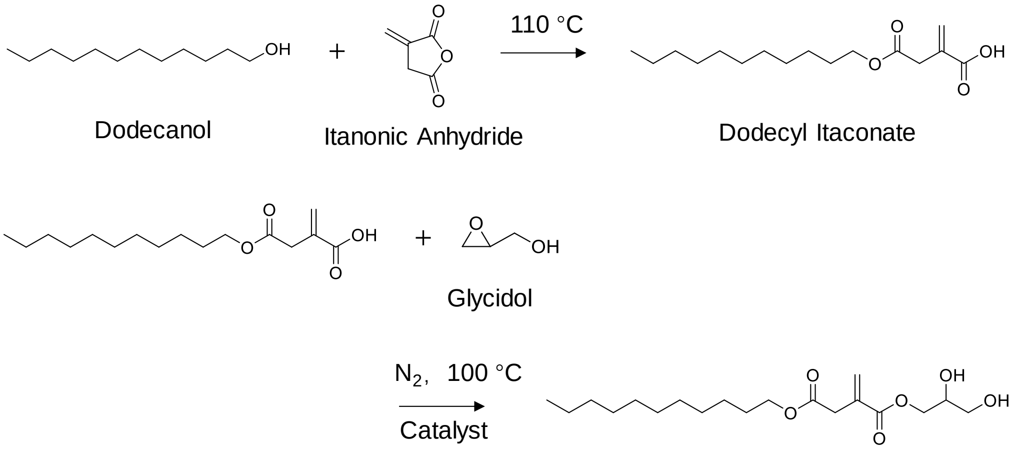

Dodecylglyceryl itaconate (DGI, n-$C_{12}H_{25}OCOCH_2C(=CH_2)COOCH_2CH(OH)CH_2OH$) was synthesized according to the literature [2-3]. Itaconic anhydride (50.0 g) was reacted with dodecanol (80.0 g) at 110 °C with stirring for 50 min. After the reaction, 100 mL hexane was added to the reaction mixture with strong stirring, and white crystals were precipitated. The pure product was recrystallized from ethanol twice and dried under vacuum. The dodecyl itaconate obtained (5.0 g, 0.017 mol) was further dissolved in toluene (5 mL), and reacted with glycidol (3.75 g, 0.051 mol) at 100 °C in the presence of 10 mg (39.7 μmol) of pyridinium p-toluenesulfonate as a catalyst. The reaction mixture was refluxed with stirring for 5 h at 100 °C. After the toluene was removed by evaporation, the crude product was obtained via silica gel column chromatography (hexane/ethyl acetate, w/w=4:6). The collected DGI fraction was purified twice by recrystallization from an acetone/hexane mixture (w/w=1 /1). ^{1}H NMR (400 MHz, DMSO-d6) δ 6.24 (s, 1H), 5.83 (s, 1H), 4.93 (d, J=5.16 Hz, 1H), 4.66 (t, J1=11.24 Hz, J2=5.64 Hz, 1H), 4.12 (d-d, J1=11.12 Hz, J2=4.32 Hz, 1H), 3.95-4.02 (m, 3H), 3.67 (m, 1H), 3.36 (m, 2H), 1.54 (m, 2H), 1.25 (m, 18H), 0.86 (t, 3H); ^{13}C NMR (100 MHz, DMSO-d6) δ 171.16, 166.53, 134.88, 129.53, 70.09, 67.04, 65.09, 63.39, 37.98, 32. 17, 29.91, 29.88, 29.83, 29.80, 29.58, 29.49, 28.92, 26.16, 22.97, 14.82.

Supplementary Methods

^{1}H NMR or ^{13}C NMR spectra: The Nuclear magnetic resonance characterization was carried out on a Bruker Advance III NMR spectrometer (Switzerland) at a resonance frequency of 400 MHz or 500 MHz. The residual protonated solvent signals were used as internal references for ^{1}H- and ^{13}C NMR spectra (^{1}H NMR: δ ($CDCl_3$). 7.26 ppm, δ (DMSO-d6). 2.50 ppm and ^{13}C NMR: δ ($CDCl_3$). 77.16 ppm, δ (DMSO-d6) 39.50 ppm. Multiplicities are abbreviated as follows: singlet (s), doublet (d), triplet (t), and multiple (m). The equilibrium photostationary state of M-azo in $CDCl_3$ ($3*10^{-3}$ M) was measured by the nuclear magnetic resonance. For detail, we prepared the sample one day before measurement and kept the sample in the dark (covered by aluminum foil). We measured the thermal stable *trans*-form of M-azo, and then irradiated the NMR tube with 365 nm light for 3 minutes. After irradiation, we immediately measured the sample. From the integration, we can calculate the *cis* content of M-azo after UV irradiation. To ensure M-azo reaches the photostationary state, we irradiated the sample and measure it again until the integration in the *cis*-, and *trans* did not change. The light in NMR room was turned off during irradiation.

Polarized optical microscopy: The images were taken using an Olympus BX51 polarized optical microscope fitted with a digital camera, a Linkam 1033L heating/cooling stage and an optical spectrometer (Ocean Optical USB2000). Images was processed using SpectraSuite and Motic Image Plus 2.2S analyze software.

3-D scanning electronic microscopy: The images were obtained using a Keyence VK-X100 laser microscope with a laser wavelength of 658 nm.

Differential scanning calorimetry: Differential scanning calorimetry (DSC) thermograms were obtained using SII Nanotechnology DSC6100. The temperature of the crucible containing the synthesized materials/film was heating and cooling at 2 °C min^{-1} or 10 °C min^{-1} with N_2 gas flow 50 mL min^{-1}. For T_g measurement in Fig. 4a, the synthesized DGI/M-azo film (before UV irradiation) was cut into small pieces with scissors and transferred into a DSC sample crucible with tweezers. The sealed sample was measured at 10 °C min^{-1} with N_2 gas flow 50 mL min^{-1} to get the DSC curve of the sample before UV irradiation. On a second day, the crucible was opened, and the small pieces of the same films (*trans*-form) were irradiated with UV light (intensity: 125 mW cm^{-2}) on both sides for 2 minutes. After irradiation, the colour of the films was changed from pale yellow (*trans*-form) to orange (*cis*-form). The orange-coloured films (*cis*-form) were transferred into another crucible and measured T_g in *cis*-form on DSC at the same condition. After measurement, the orange-colored film recovered to pale yellow color, indicating the *cis*-form was thermal isomerized into *trans*-form during heating.

UV–Vis absorption spectra: The spectrum was collected using a JASCO V-670 spectrophotometer (JASCO). For solution state, M-azo compounds were dissolve in $CHCl_3$ (~$5*10^{-5}$ M). The measurements were made using double-beam spectrophotometer with 1-cm quartz cuvettes and $CHCl_3$ as the blank. The polymer film (5 μm) was on a thin quartz glass and using the glass as blank. The sample was irradiated with UV or Visible light for different times. The irradiation time is automatically controlled by the light source. The light source is using high-power mercury light

(REX-250) with different filters.

X-ray diffraction: X-ray diffraction data for the free-standing film were collected on a SmartLab Rigaku-X-ray analytical machine with Cu Kα (λ=1.5418 Å). The measurement was carried out using an X-ray generator with a voltage of 45 kV and a current of 120 mA. The scan runs at a step size of 0.01°2θ. The sample was directly placed on the detecting substrate. X-RD data were collected for the same polymer after UV irradiation at the same scanning conditions.

Fourier transform infrared spectroscopy: The FT-IR transmission spectra were obtained using a Perkin-Elmer Spectrum 2000 spectrometer. The samples were analyzed in transmission mode. M-azo compound was measured by casting M-azo compound in $CHCl_3$ solutions on KBr tablets. The free-standing film was fixed on the sample stage and analysis before UV and after UV irradiation.

Supplementary Figures

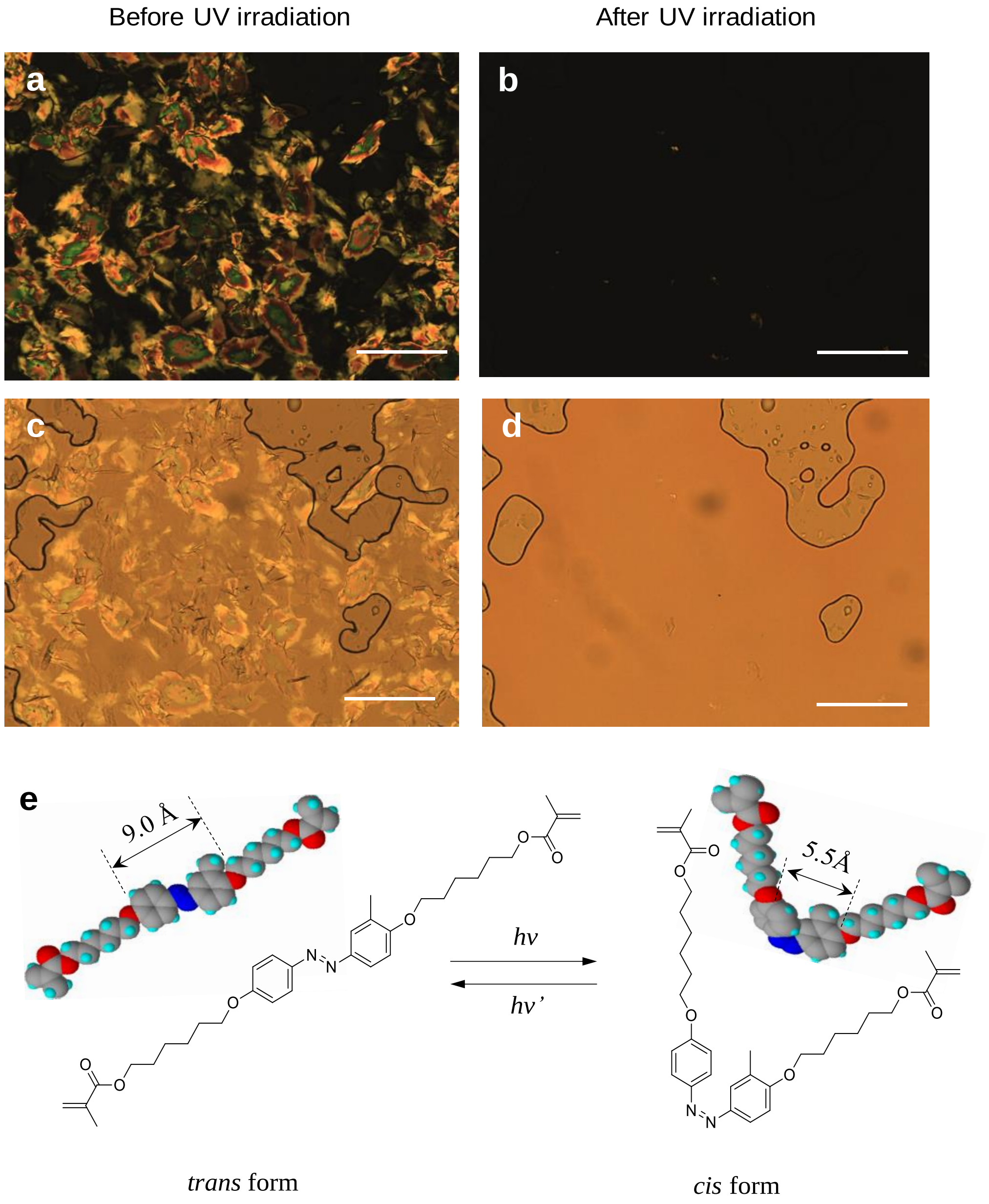

Supplementary Figure 1. The detection of M-azo molecules' phase transition under polarized optical microscopy (POM). (**a**, **b**) POM images and (**c**, **d**) microscopy images of the compound on irradiation by UV light (λ= 365 nm). The UV irradiation induced a quick (a) anisotropic to (b) isotropic phase, and the corresponding (c) solid to (d) liquid phase transition. (**e**) The geometrical conversion of M-azo from extended *trans* to *cis*-form with a molecular length change from 9 to 5.5 Å upon light irradiation. Scale bars, 100 μm.

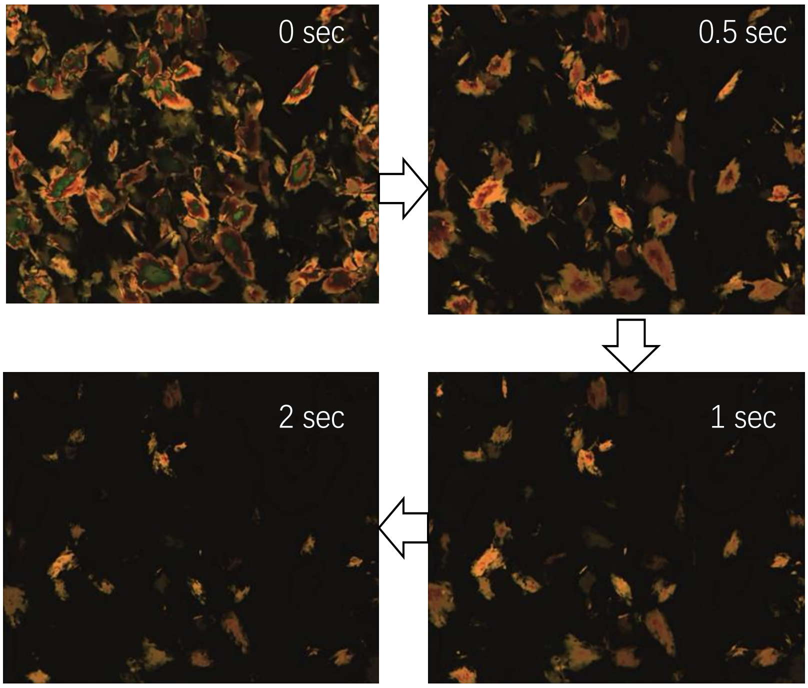

Supplementary Figure 2. The POM detection of quick photomelting process of M-azo under UV irradiation. The birefringence of the M-azo under POM disappeared quickly, changing from crystal phase to isotropic phase.

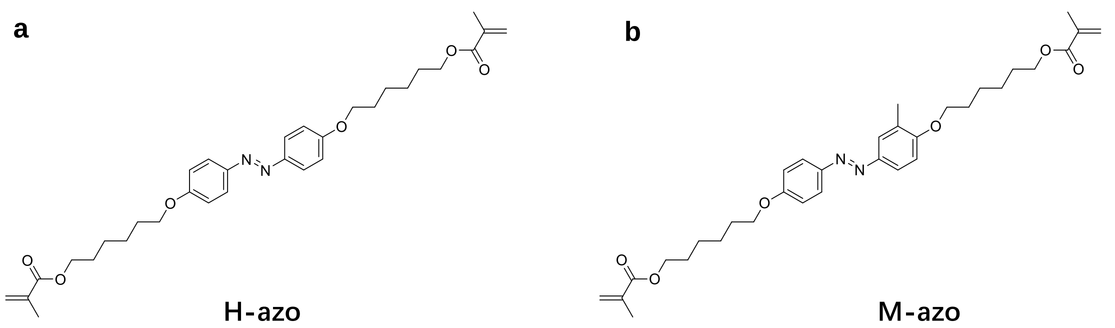

Supplementary Figure 3. The chemical structures of H-azo and M-azo. (**a**) H-azo. (**b**) M-azo.

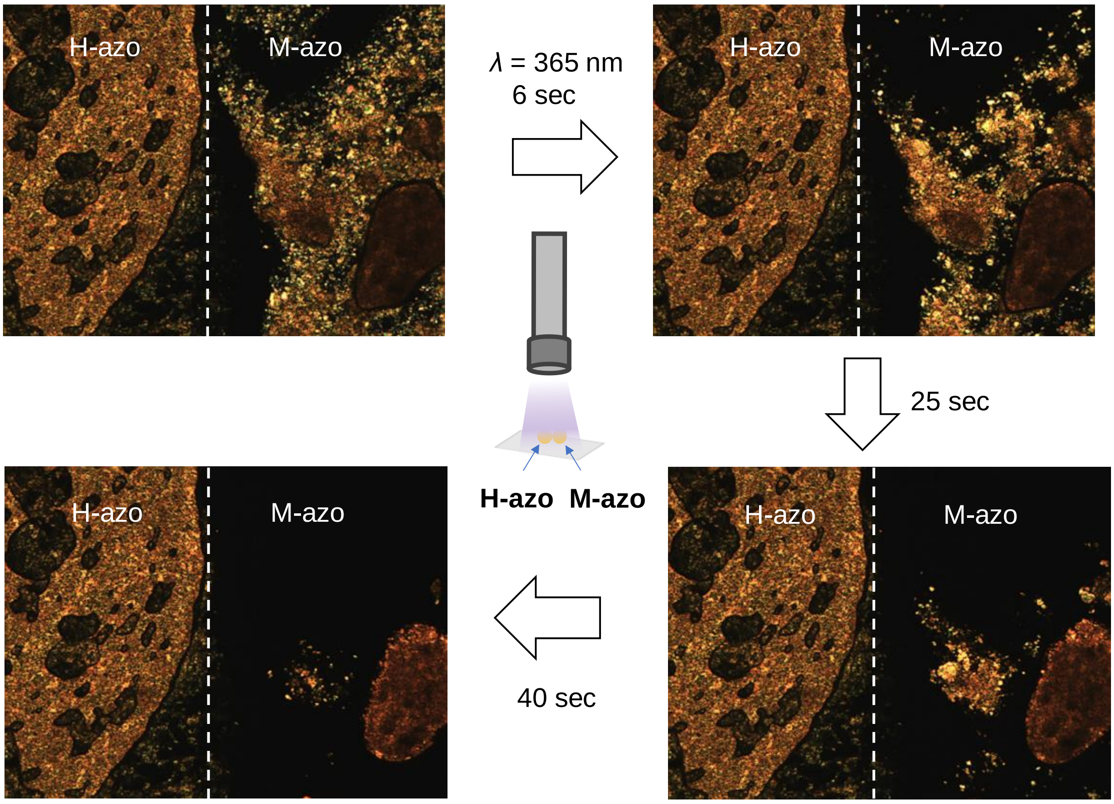

Supplementary Figure 4. Difference in the photomelting properties of M-azo and H-azo placed together on a slide glass under POM. In contrast with H-azo showing no photomelting, M-azo shows a fast photo-induced phase transition although there is only slight difference in their molecular structures.

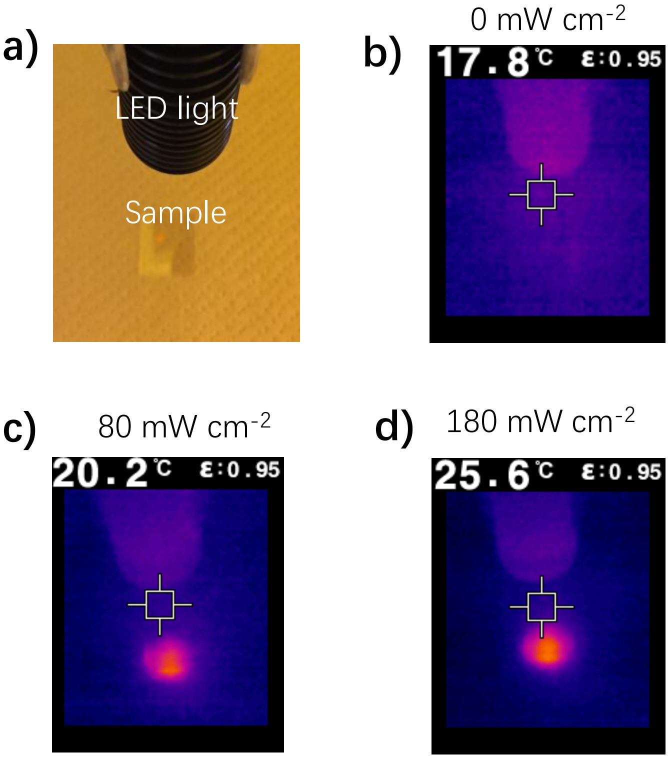

Supplementary Figure 5. Surface temperature of the compounds (M-azo) before and after UV irradiation measured using an infrared thermometer. (**a**) Photograph of the sample and LED light. (**b-d**) Infrared thermometer images of the sample under irradiation with 365 nm light for 10 s with different intensities.

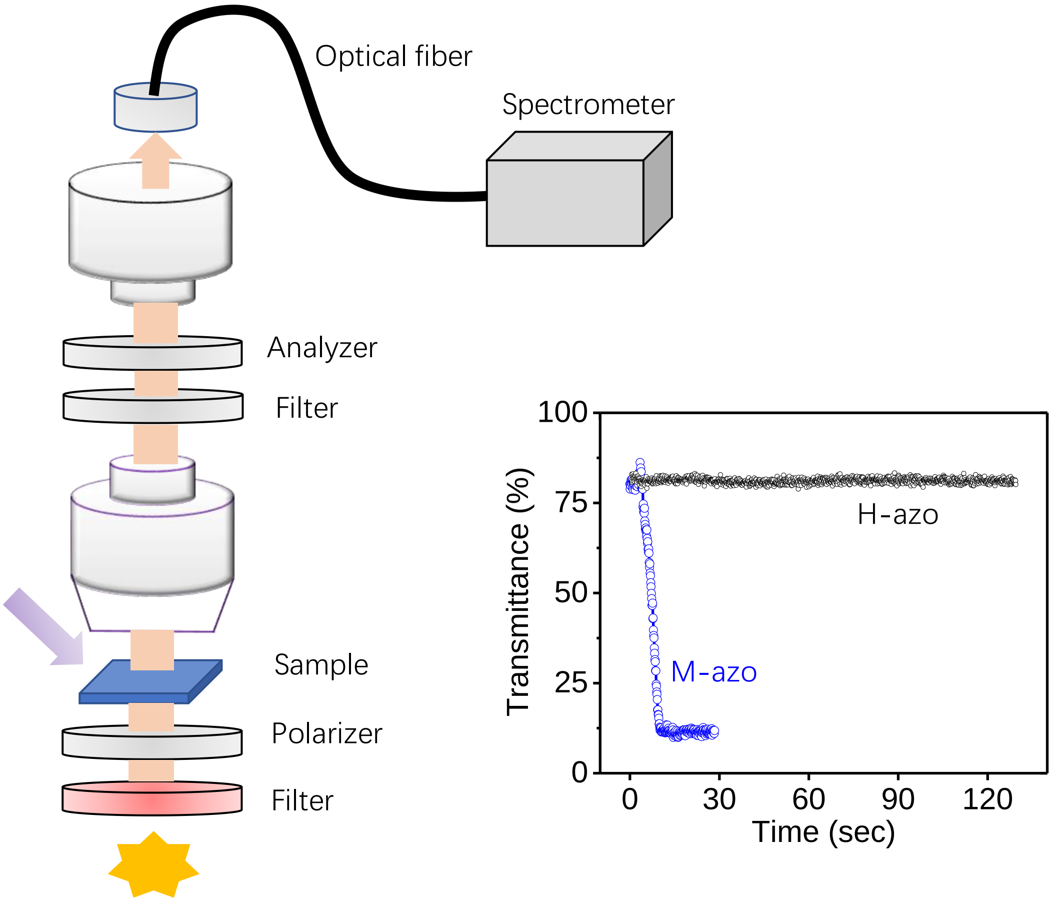

Supplementary Figure 6. Evaluation of kinetics of photo-induced phase transition for M-azo and H-azo. The experimental set-up is shown on the left. We optically measured the extent of the photoinduced solid to liquid phase transition of M-azo and H-azo by monitoring the light intensity of 650 nm transmitted through a sample which is placed between two polarizers at crossed configuration. The M-azo or H-azo compounds were sandwiched between two cover glasses and placed on the polarized optical microscope. When the sample was irradiated with UV light from an ultra-high mercury light (REX-250, intensity: 125 mW cm^{-2}), the transmitted light at 650 nm was immediately monitored by an Ocean Optics USB 2000 spectrometer.

The data is shown on the right. During the monitor time (~180 s), the M-azo decrease the transmittance because M-azo melt during UV irradiation. The H-azo shows no decrease in the transmittance during the detecting time (~180 s) because UV light cannot melt H-azo.

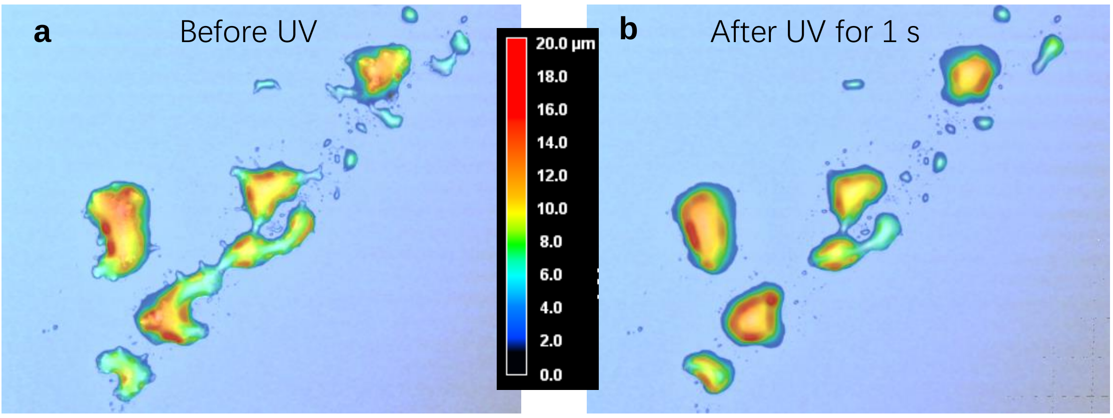

Supplementary Figure 7. The 3D scanning electronic microscopy shows the UV irradiation induced a solid-to-liquid change of M-azo. (**a**) M-azo before UV irradiation. (**b**) M-azo after UV irradiation.

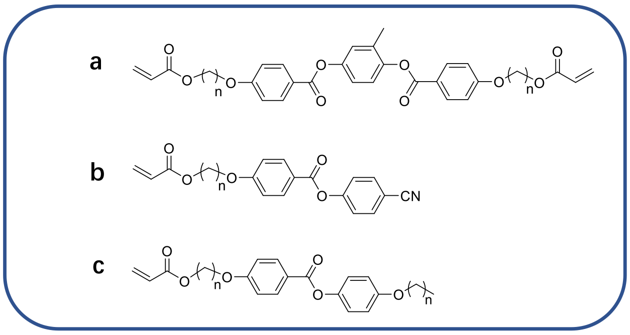

Year	Journal	Monomer used
2008	Macromolecules 2008, 41, 8592-8596	a, c
2009	Nature materials, 2009, 8, 677-682	a, b, c
2009	J. Mater. Chem., 2009, 19, 1080-1085	a
2011	Adv. Funct. Mater. 2011, 21, 2913-2918	a
2012	Angew. Chem. Int. Ed., 2012,51,4644-4647	a, b, c
2012	Advanced materials, 2012, 24, 2839-2843	a
2012	Angew. Chem. Int. Ed, 2012, 51, 12469-12472	a, b, c
2012	Angew. Chem. Int. Ed. 2012, 51, 892–896	a, b, c
2013	Advanced materials, 2013, 25, 5880-5885	a
2014	Nature chemistry, 2014, 6, 229-235	a, b, c
2015	Nature Communications, 2015, 6, 8334-8340	a, b, c
2015	Science, 2015, 347, 982-984	a
2016	Soft matter, 2016, 12, 3196-3201	a, c
2017	Advanced materials, 2017, 29, 1606712-1606717	a, b
2017	Angew. Chem. Int. Ed., 56, 1-5	a, b
2017	Nature, 2017, 546, 632-636	a, b

Supplementary Table 1. Nematic liquid-crystalline monomers that typically used in photoresponsive polymers in literature. As shown in the above, there are limited non-azobenzene LC monomers (mainly three types: a, b, c) which are used in LC photoresponsive polymers for the past ten years. In this work, we developed a polymerizable smectic LC monomer, DGI, as a new candidate for application in photoresponsive polymers. Compared with the above there nematic LC monomers (commercial available), DGI is easy to synthesize and has no obvious absorption between 300-600 nm.

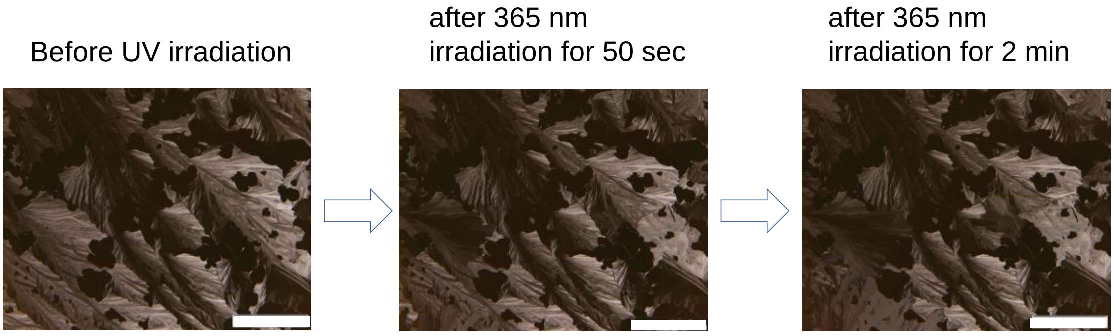

Supplementary Figure 8. The POM images of DGI compounds at UV (λ = 365 nm) irradiation for different time. DGI compounds shows no photo-responsibility (light intensity: 100 mW cm^{-2}). Scale bars, 200 μm.

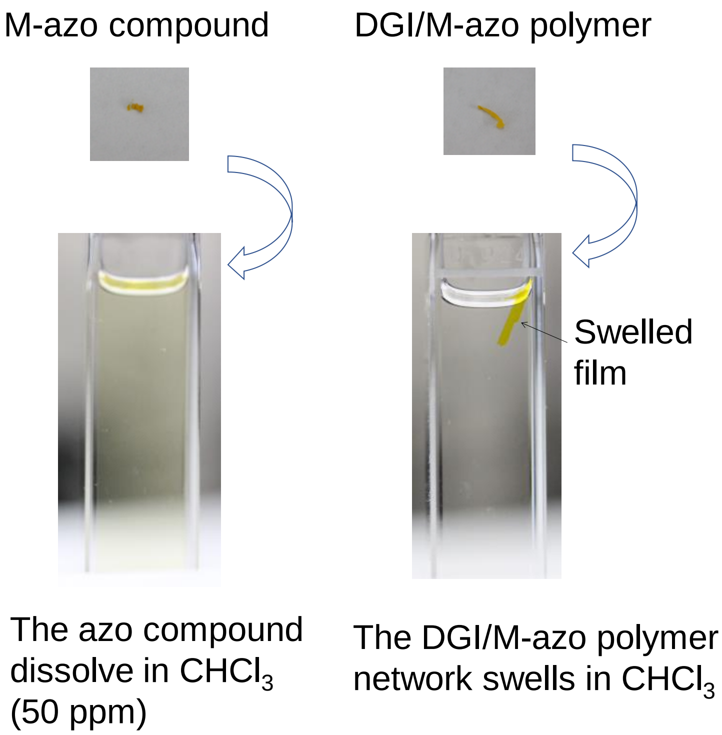

Supplementary Figure 9. An experiment shows that the M-azo is crosslinked in the polymer network. On the left: when ~0.0001 g M-azo compounds was dissolved in 3 mL $CHCl_3$, the solution changed color from transparent to yellow with a very low concentration of 50 ppm. On the right: a small DGI/M-azo film was swelled in $CHCl_3$. This experiment indicates that M-azo is covalently crosslinked in DGI/M-azo film. The polymerization condition has achieved a full conversion of the acrylate monomers.

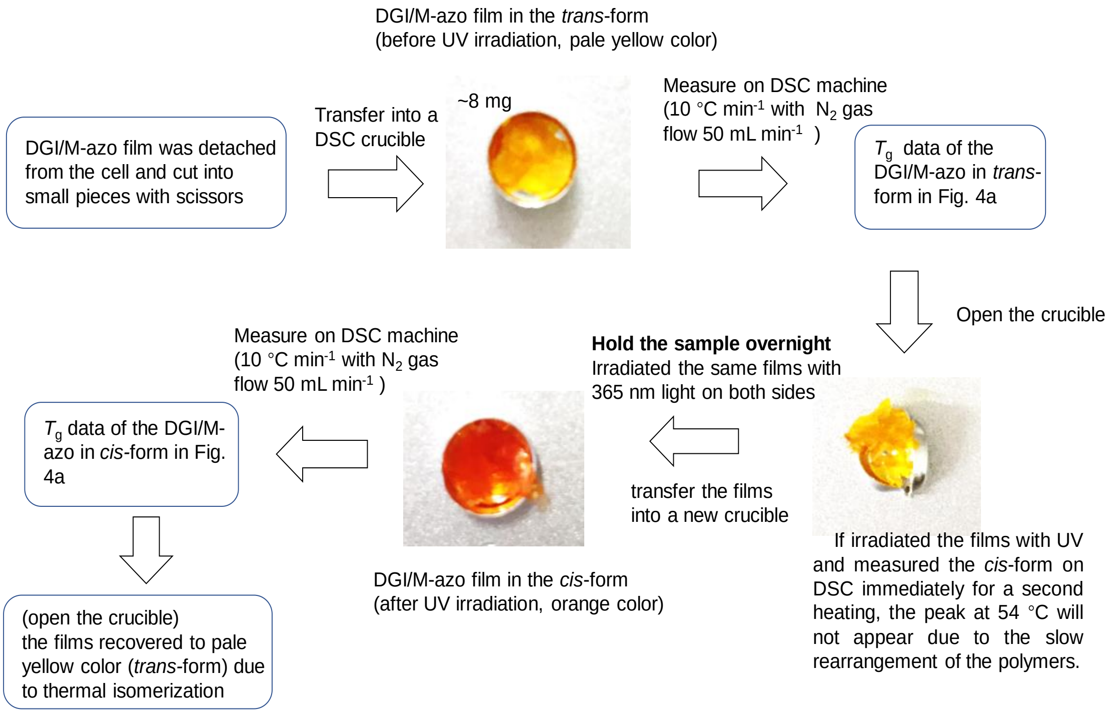

Supplementary Figure 10. The detail process on T_g measurement of the *trans*-, and *cis*-film. A prepared DGI/M-azo film (5 μm) shows pale yellow color before UV irradiation (*trans*-form, thermal stable state). The T_g value in the *trans*-form can be measured directly on DSC. To measure the *cis*-form, UV irradiation is needed (*trans*-to-*cis* photoisomerization). When the films were irradiated with UV light (intensity = 125 mW cm^{-2}), they changed color from pale yellow (*trans*-form) to orange (*cis*-form).

During UV irradiation and sample transfer into the crucible, the light in the lab was turned off (or using light with <500 nm cut off) because the lab light may also return the *cis*-azo to *trans*-form. After sufficient UV irradiation, the film reached a photostationary state (Supplementary Figure 14). Then the T_g were measured on DSC of the film in the *cis*-form.

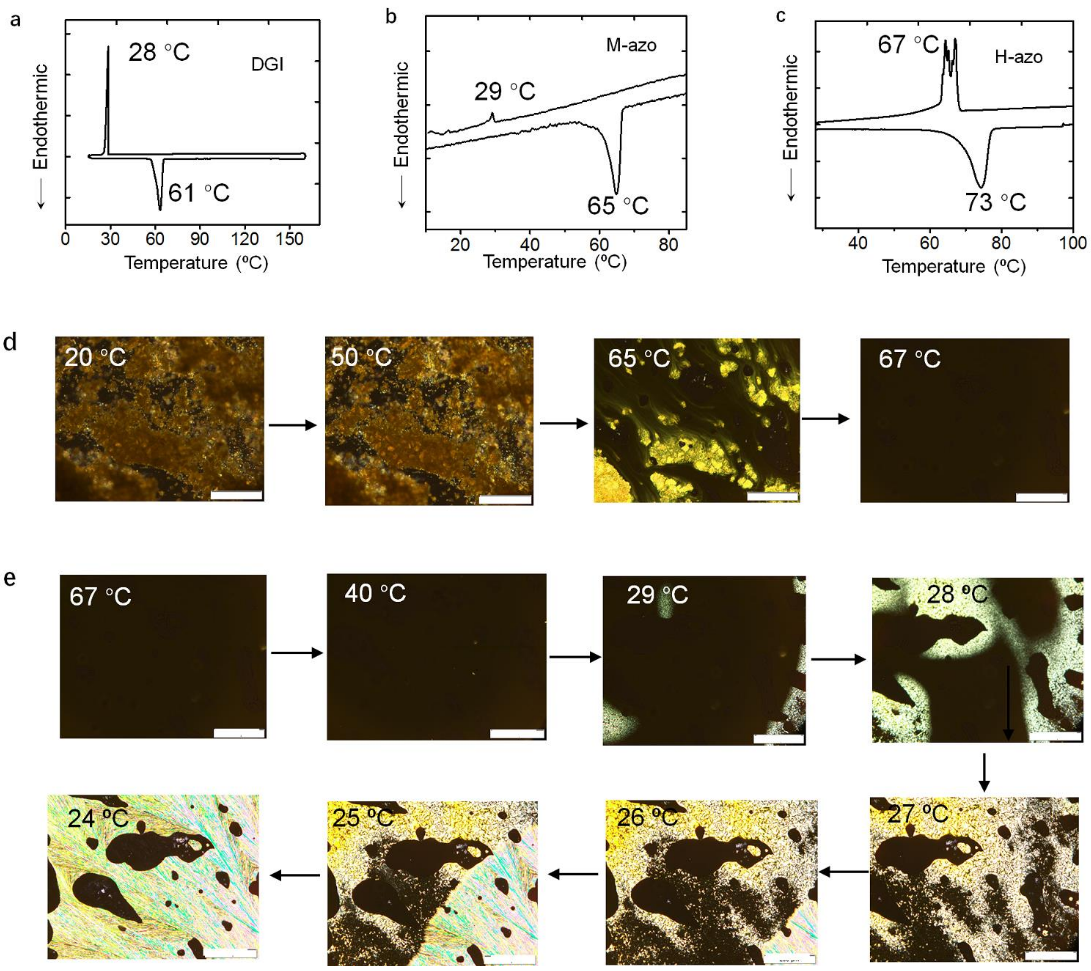

Supplementary Figure 11. Differential scanning calorimetry (DSC) curves and POM images of M-azo under heating and cooling. DSC curves of (**a**) DGI, (**b**) M-azo and (**c**) H-azo. The temperature of the crucible containing the compounds was heating and cooling at 2 °C min^{-1} with N_2 gas flow 50 mL min^{-1}. For M-azo, the upper line has a small peak, but the bottom line has a strong peak. This is related to the phase transition behaviors of M-azo observed from the POM during heating (**d**) and cooling (**e**). Scale bars, 200 μm.

At 65°C, M-azo quickly melted. The POM images become dark within 2-4 s. During cooling, M-azo begin to nucleation (partial) and crystalize at 29 °C. This process is relatively slow and changed gradually, which resulted in the difference from M-azo DSC data between heating and cooling process. The heating and cooling on the POM was controlled by a heating/cooling stage Linkam 1033L at a speed of 5 °C min^{-1}.

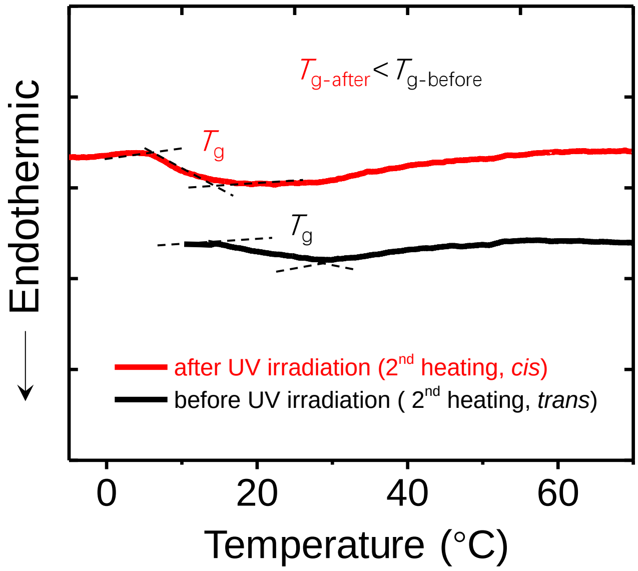

Supplementary Figure 12. The DSC curves of the DGI/M-azo film before and after UV irradiation at 2nd heating. The phase transition peak at 54 °C was not detected on the second heating (either for the *trans*- or *cis*-sample), which was carried out immediately after first heating and cooling. For the *cis*-sample, UV irradiation was done at the beginning of the second heating.

This peak at 54 °C appeared again after holding the sample for overnight, suggesting that the rearrangement of the polymer chains is slow[4]. At the first heating to 70 °C, the ordered structure in the polymer was destroyed. During cooling process, the polymers began to rearrange.

The cooling time of the DSC measurement is about 15-60 minutes depending on the cooling speed (10 °C min^{-1} or 2 °C min^{-1}). Within the testing time (15-60 min), DGI/M-azo polymer has not enough time to rearrange and reorder, so the peak at 54 °C will not appear on the second heating if measure immediately. However, after holding the sample for overnight, this peak appeared again because overnight was enough time for the polymer to rearrange. $T_{g\text{-before}}$: glass transition temperature before UV irradiation; $T_{g\text{-after}}$: glass transition temperature after UV irradiation.

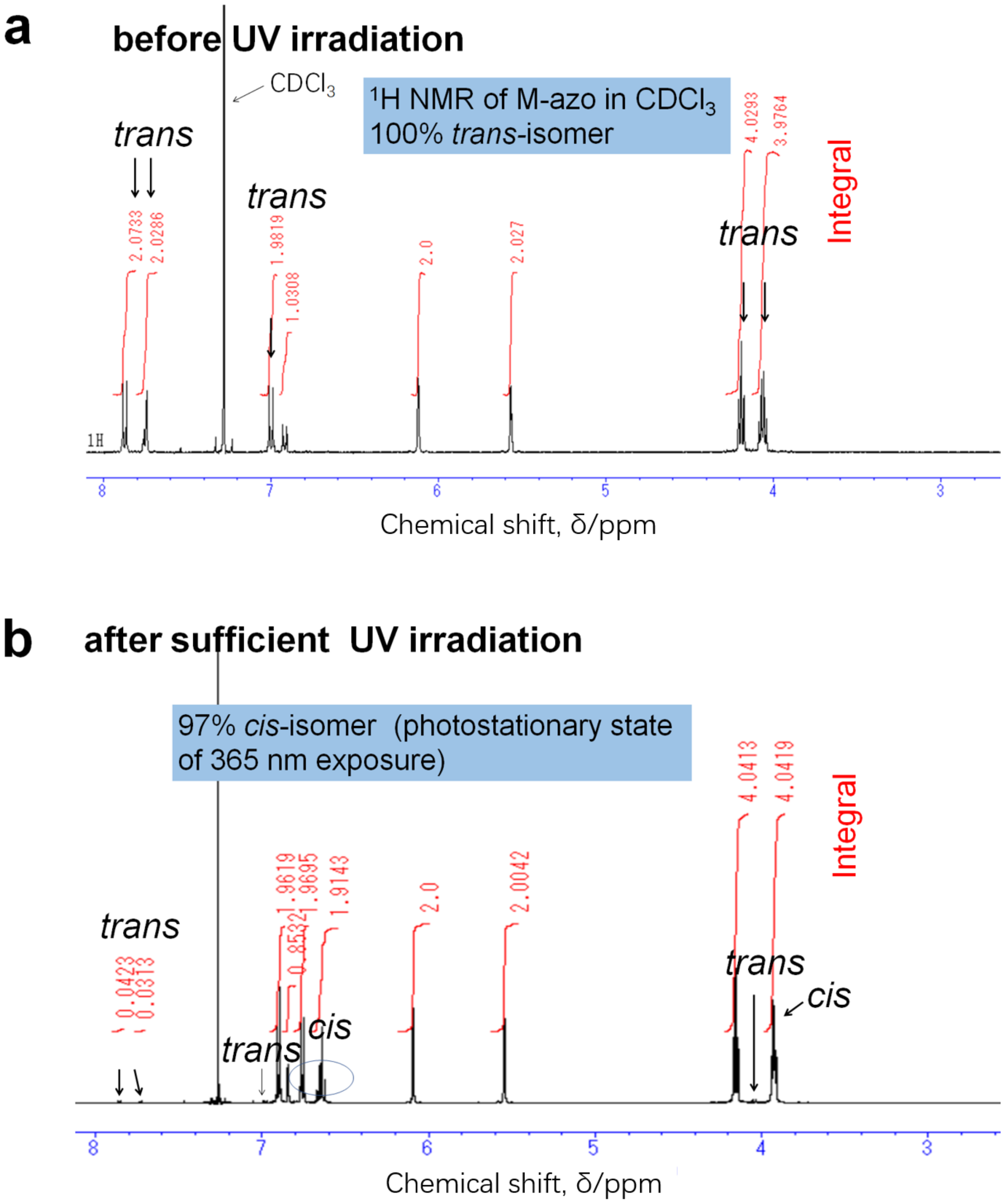

Supplementary Figure 13. ^{1}H NMR spectra of M-azo in $CDCl_3$ before and after 365 nm irradiation. (**a**)100% *trans*-azo before UV irradiation; (**b**) Photostationary state of M-azo in $CDCl_3$ (~$3*10^{-3}$ M). The equilibrium photostationary state of M-azo is achieved by continues 365 nm light irradiation (intensity: 125 mW cm^{-2}) until the integration from the spectrum (bottom) shows no further change. The integration in the spectrum shows that approximately 97% of the *trans* isomers was converted to *cis* isomers

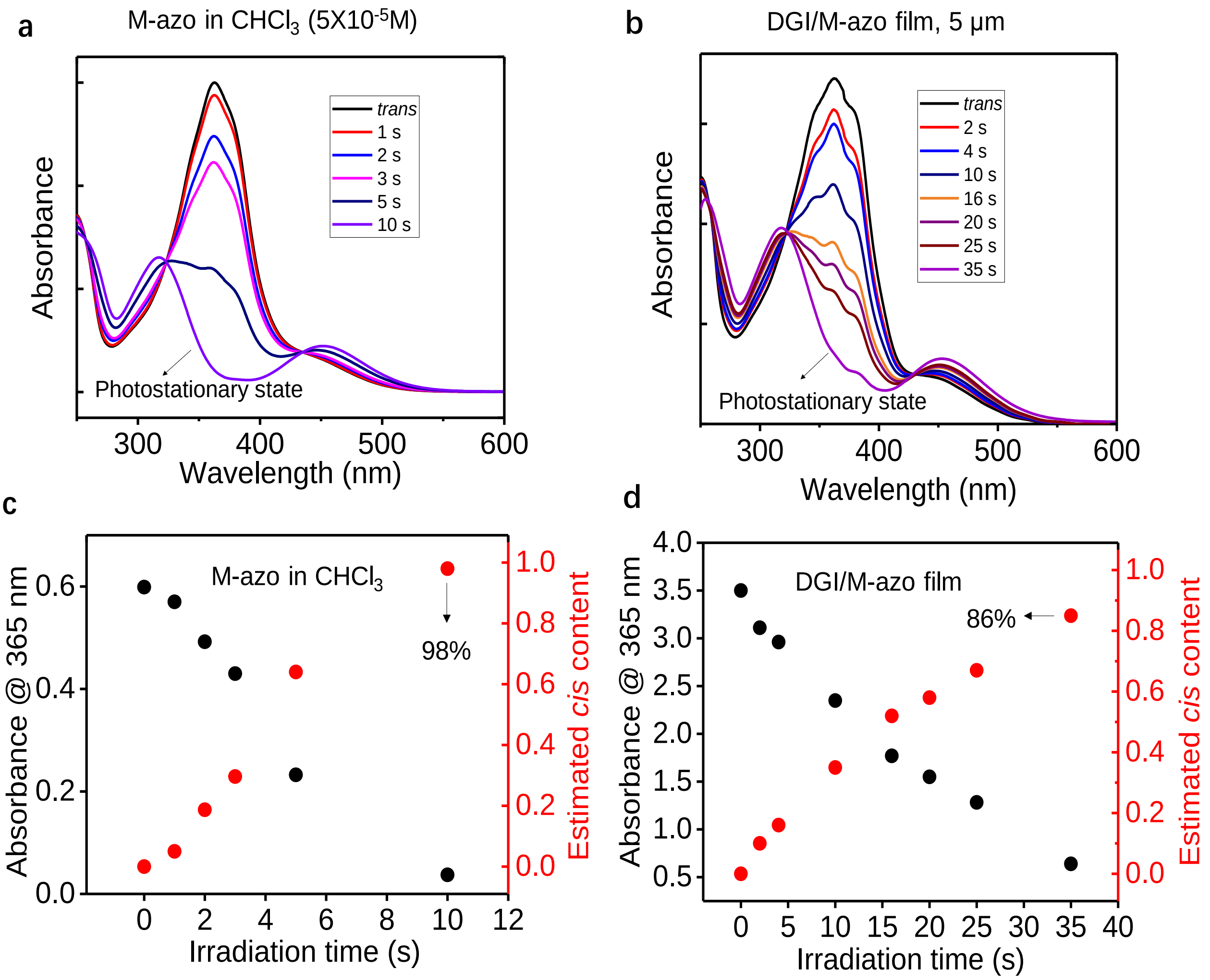

Supplementary Figure 14. *Cis*-contents of M-azo in the solution and film state. (**a**) UV-vis absorption spectra of M-azo (in $CHCl_3$, ~5*10^{-5}M) before UV (λ = 365 nm, intensity = 40 mW cm^{-2}) (*trans*-isomers) and after UV irradiation for different times. (**b**) UV-vis absorption spectra of DGI/M-azo film (5μm) before UV (λ = 365 nm) and after UV irradiation after different time. (**c**) Absorbance at 365 nm and the estimated *cis* content in (a) as a function of time. d. Absorbance at 365 nm and the estimated *cis* isomers in (b) as a function of time. The *cis* content was estimated by a method in literature[5-6]:

Cis content = (1-A/A $_{trans}$)/(1-$\mathcal{E}_{cis}/\mathcal{E}_{trans}$), where A is the absorbance of M-azo in $CHCl_3$ or DGI/M-azo film, A $_{trans}$ is the absorbance of *trans* in solution or film state, and $\mathcal{E}_{cis}/\mathcal{E}_{trans}$ = ~0.05 for azobenzene[5-6]. The estimated *cis* content of M-azo in $CHCl_3$ in the photostationary state is 98%, which is close to the value, 97%, that calculated from the integration in the ^{1}H NMR spectrum in Supplementary Figure 13. In the film state, the estimated *cis* content is about 86% after UV irradiation.

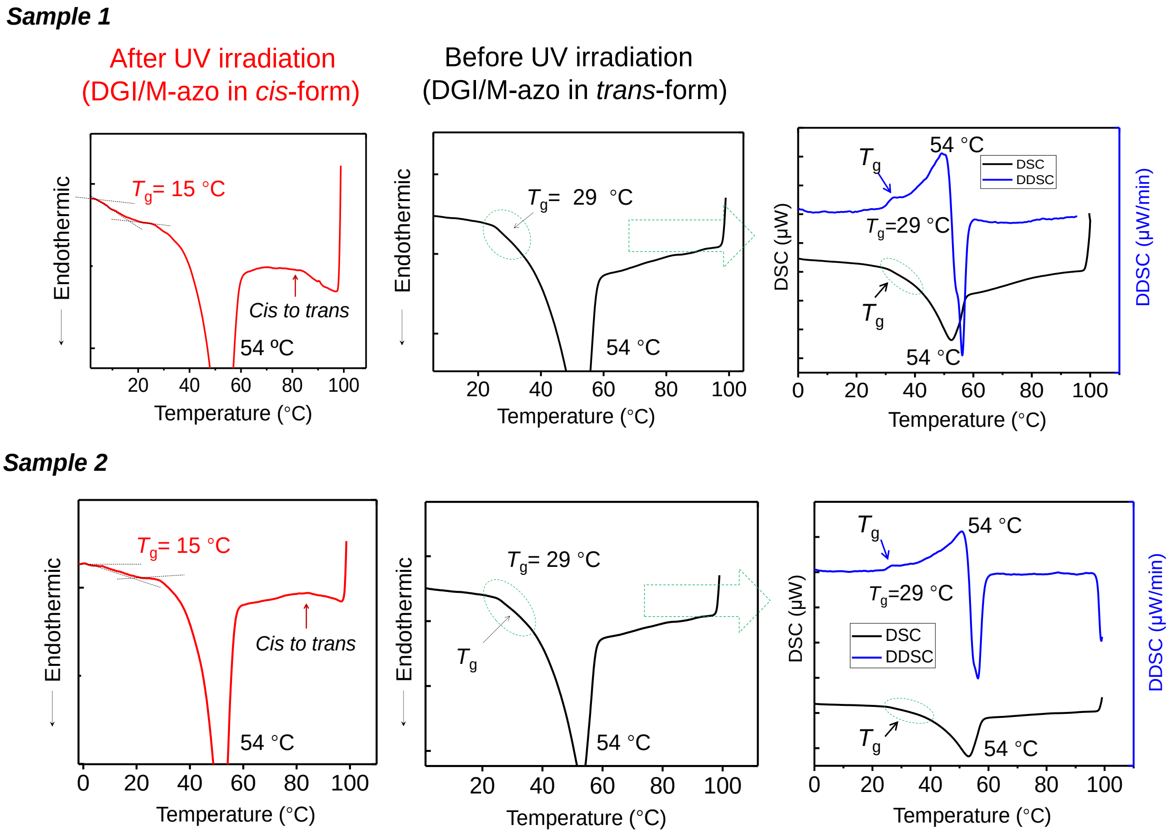

Supplementary Figure 15. Differential scanning calorimetry (DSC) first heating curves of the DGI/M-azo film in *cis*-, *trans*-form. The DGI/M-azo polymer films (sample 1 and sample 2) show lower T_gs (~15 °C) in the *ci*s-form and a higher T_gs (~29 °C) in the *trans*-form. Due to the big endothermic peak of DGI mesogens at 54 °C, T_g in *trans*-form is sometimes not obvious from DSC curve. When T_g is difficult to determine for *tran*s-form on DSC, the derivative signal, DDSC (d Q/dT vs. T) (where Q is the heat flow) can help, because the heat capacity increase will be replaced by a peak from DDSC (arrow on blue line)[7].

Cis-to-*trans* isomerization releases heat. For the *cis*-sample (after UV irradiation), there was a broad exothermic peak due to the thermal *cis*-to-*trans* isomerization. The *cis*-to-*trans* exothermic band (arrow on red line) in our sample appears at the temperature range from 80 to 100 °C and they are not obvious if the DSC curve is not enlarged. This is probably because the azo content in the film is relatively low.

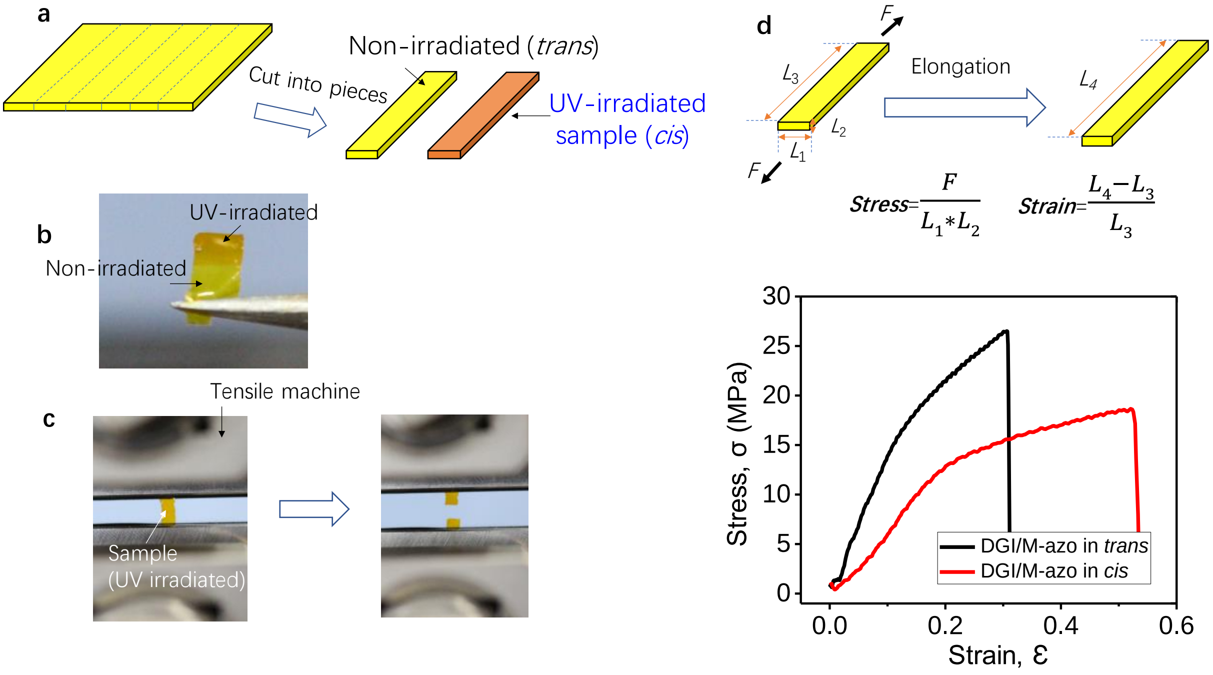

Supplementary Figure 16. Stress-strain curves of DGI/M-azo films before (*trans*-form) and after UV irradiation (*cis*-form). (**a**) the tested sample were cut into pieces with knifes. (**b**) the photo shows a different color in the UV irradiated (*cis*-form) and non-irradiated regions (*trans*-form). (**c**) test the samples upon uniaxial stretching on a tensile machine at room temperature. (**d**) the stress-strain curves of the samples in the *trans* and *cis* form.

Young's modulus Hook's law assumes perfect elasticity in a material body[8]. Young's modulus, E=σ/Ɛ, where σ and Ɛ represent the tensile stress and strain[8]. It is a fundamental measure of the stiffness of the material and was evaluated as a ratio of stress to strain in a small strain limit[8].

The DGI/M-azo film in the *trans*-form showed higher Young's modulus of 140.0 MPa, while the film in the *cis*-form showed a lower Young's modulus of 65.6 MPa. This is consistent with the results that the *trans*-form of DGI/M-azo film has a higher T_g, and the *cis*-form has a lower T_g. The thickness of the film 10 μm. Crosslinker: ~40wt%. Testing temperature: ~20 °C.

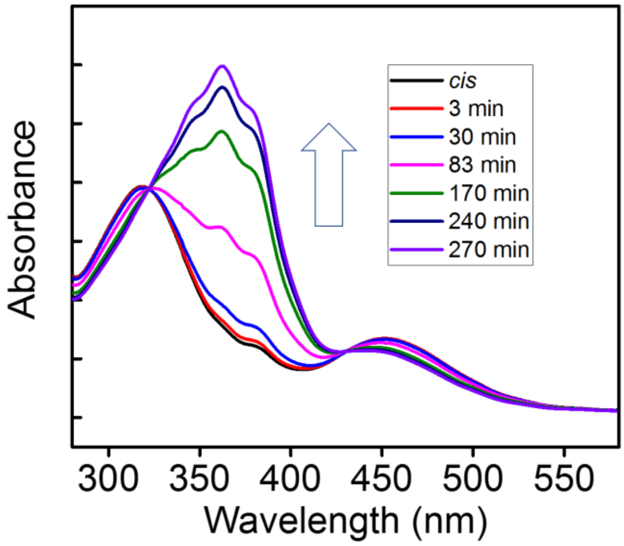

Supplementary Figure 17. UV-vis absorption spectra of thermal relaxation of the *cis* state to *trans* state of DGI/M-azo film measured at room temperature in the dark. The relaxation in the dark back to *trans* takes many hours with ~80% conversion of 6 h. This experiment also indicates the life-time in the *cis* state of M-azo covalently in the film is long enough for us to measure the T_g on DSC.

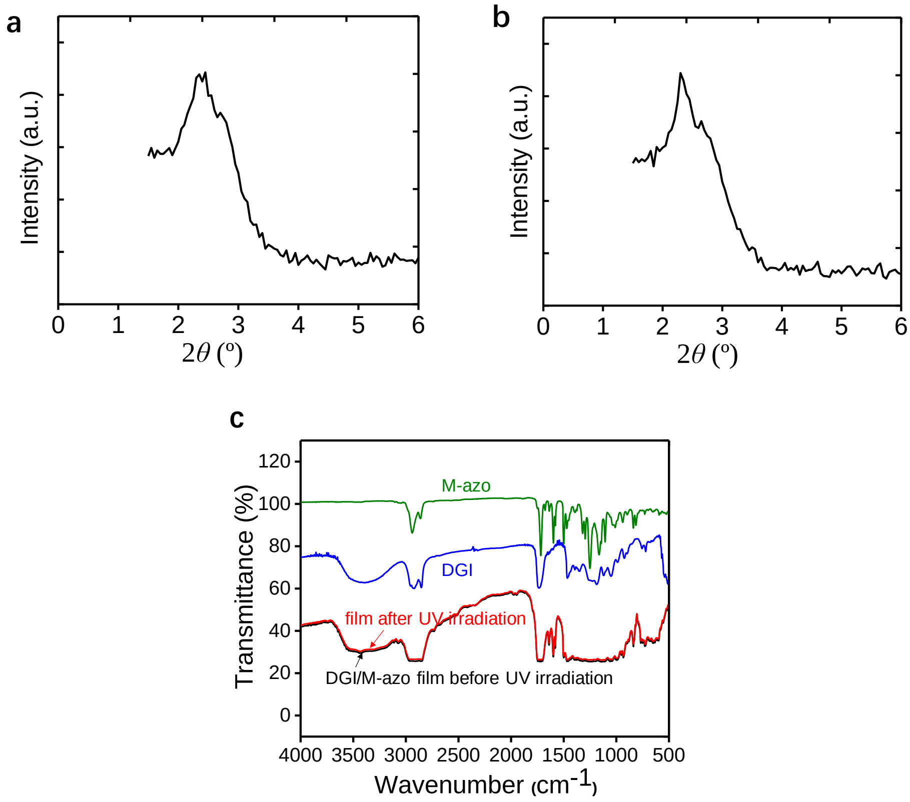

Supplementary Figure 18. Structural characterizations of the same film before and after UV irradiation. The X-ray diffraction pattern of the free-standing film at room temperature (**a**) before and (**b**) after UV irradiation. (**c**) FT-IR spectrum of the film before and after UV irradiation.

Supplementary References

1. S. Muhammed, O. Jesper, T. Helena, S. Kent, K. Mikhail, Synthesis, spectroscopic characterization and alignment of novel azobenzene-containing monomers. Liquid Crystals, **32**, 901-908, (2005).
2. K. Naitoh, Y. Ishii, K. Tsujii, Iridescent phenonmena and polymerization behaviors of amphiphilic monomers in lamellar liquid crystalline phase. J. Phys. Chem. **95**, 7915-7918, (1991).
3. K. Tsujii, M. Hayakawa, T. Onda, T. Tanaka, A novel hybrid material of polymer gels and bilayer membranes. *Macromolecules* **30**, 7397-7402, (1997).
4. Tashiro *et al*. Structure and thermochromic solid-state phase transition of poly (3-alkylthiophene), Journal of Polymer Science: Part B: Polymer Physics, **29**, 1223-1233, (1991).
5. J. G. Victor, J. M. Torkelson, On Measuring the Distribution of Local Free- Volume in Glassy-Polymers by Photochromic and Fluorescence Techniques. Macromolecules **20**, 2241-2250, (1987).
6. H. Zhou, *et al*. Photoswitching of glass transition temperatures of azobenzene-containing polymers induces reversible solid-to-liquid transitions. Nat. Chem. **9**, 145-151, (2017).
7. J. D. Menczel, R. B. Prime, Thermal analysis of polymers: fundamentals and application, John Wiley & Sons, Inc, 2009.
8. L. H. Sperling, Introduction to physical polymer science, 4th ed, John Wiley & Sons, Inc, 2006.
