# Supplementary material for: Light-induced mechanical response in crosslinked liquid-crystalline polymers with photoswitchable glass transition temperatures
Source: Nat Commun. 2018 Aug 13;9:3234. doi: 10.1038/s41467-018-05744-x (PMC6089925; doi:10.1038/s41467-018-05744-x)
Supplement: Supplementary file 2 — Description of Additional Supplementary Files [file 41467_2018_5744_MOESM2_ESM.pdf]

Description of Additional Supplementary Files

File Name: Supplementary Movie 1
Description: The difference in the photo-induced phase transition behaviors of M-, and H-azo.

File Name: Supplementary Movie 2
Description: The continues photomechanical response of the DGI/M-azo film under light.

File Name: Supplementary Movie 3
Description: The bending of the polymer in response to a UV light.
